# Harnessing Magnetic Nanoparticles for the Effective Removal of Micro- and Nanoplastics: A Critical Review

**DOI:** 10.3390/nano14141179

**Published:** 2024-07-11

**Authors:** Sabina Vohl, Matjaž Kristl, Janja Stergar

**Affiliations:** Faculty of Chemistry and Chemical Engineering, University of Maribor, Smetanova 17, 2000 Maribor, Slovenia; sabina.vohl@um.si (S.V.); matjaz.kristl@um.si (M.K.)

**Keywords:** magnetic nanoparticles, microplastics, nanoplastics, water treatment, removal

## Abstract

The spread of micro- (MPs) and nanoplastics (NPs) in the environment has become a significant environmental concern, necessitating effective removal strategies. In this comprehensive scientific review, we examine the use of magnetic nanoparticles (MNPs) as a promising technology for the removal of MPs and NPs from water. We first describe the issues of MPs and NPs and their impact on the environment and human health. Then, the fundamental principles of using MNPs for the removal of these pollutants will be presented, emphasizing that MNPs enable the selective binding and separation of MPs and NPs from water sources. Furthermore, we provide a short summary of various types of MNPs that have proven effective in the removal of MPs and NPs. These include ferromagnetic nanoparticles and MNPs coated with organic polymers, as well as nanocomposites and magnetic nanostructures. We also review their properties, such as magnetic saturation, size, shape, surface functionalization, and stability, and their influence on removal efficiency. Next, we describe different methods of utilizing MNPs for the removal of MPs and NPs. We discuss their advantages, limitations, and potential for further development in detail. In the final part of the review, we provide an overview of the existing studies and results demonstrating the effectiveness of using MNPs for the removal of MPs and NPs from water. We also address the challenges that need to be overcome, such as nanoparticle optimization, process scalability, and the removal and recycling of nanoparticles after the completion of the process. This comprehensive scientific review offers extensive insights into the use of MNPs for the removal of MPs and NPs from water. With improved understanding and the development of advanced materials and methods, this technology can play a crucial role in addressing the issues of MPs and NPs and preserving a clean and healthy environment. The novelty of this review article is the emphasis on MNPs for the removal of MPs and NPs from water and a detailed review of the advantages and disadvantages of various MNPs for the mentioned application. Additionally, a review of a large number of publications in this field is provided.

## 1. Introduction

Microplastics (MPs) and nanoplastics (NPs) in water represent a global environmental challenge with numerous adverse effects on aquatic ecosystems and potentially on human health. MPs refer to small plastic particles measuring less than 5 mm, while the environmental breakdown of MPs leads to NPs, which are even smaller particles that can be just a few nanometers in size (≤100 nm) [1]. NPs are widespread pollutants found in various environmental media such as soil, seas, freshwater, and even frigid zones. Their small size and properties allow them to permeate biological membranes, leading to significant toxicity in organisms throughout the food chain. The production levels of thermoplastics like high-density polyethylene (HDPE), low-density polyethylene (LDPE), polypropylene (PP), polystyrene (PS), polyvinyl chloride (PVC), and polyethylene terephthalate (PET) are reflected in the degree of MP contamination found in freshwaters and drinking water: PE ≈ PP > PS > PVC > PET [2]. Removing NPs from water is a crucial challenge. Research has focused on understanding the effects of NPs on aquatic macrophytes, including their absorption, adsorption, and toxicological consequences. It is important to note that plants and aquatic systems are essential for the human diet and have implications for food security and safety. The reported effects of NPs include reproductive toxicity, growth inhibition, oxidative damage, inflammation, immunotoxicity, and behavioral changes. NP particles pose significant concerns in ecosystems due to their short-term and long-term effects on organisms, and they often escape common elimination methods due to their small size. Therefore, a critical assessment of their sources, transport, impacts, and removal technologies is necessary to address the research issues associated with NP pollution and remediation in aquatic ecosystems [3]. Various methods have been employed for the removal of NPs from water, but some have limitations in terms of minimum particle size and suitability for laboratory analysis. Purification and isolation methods such as filtration, evaporation, solvent extraction, density separation, and gravitational separation exist, but each method has shortcomings due to the unique properties of NPs. Membrane filtration works for smaller volumes but has a slow flow rate and can lead to clogging and particle adherence to filters, reducing detection. Density separation using saturated salt solutions has been successful, but is not widely used due to challenges in collecting nanoparticles from the liquid–air interface. Previous reports may have underestimated NPs’ abundance due to difficulties in the detection and removal of smaller particles. Adsorption methods are commonly used to remove various pollutants from water and offer benefits such as cost-effectiveness, ease of incorporation, and simplicity [4]. Iron oxide nanoparticles (IONPs) possess a large surface area per unit of mass, making them effective for adsorption. This characteristic has led to their utilization in various applications, including the removal of environmental pollutants. MNPs play a crucial role in various environmental and energy sustainability processes. For instance, magnetic chitosan microspheres chemically modified with thiourea for metal ions’ adsorption [5], magnetic tubular nanofibers for the removal of uranium, copper, and mercury from wastewater [6,7,8], magnetic hyper-cross-linked polymers for antibiotics’ removal from water [9], and magnetic hexacyanoferrate material for cesium sorption from water [10]. The magnetic properties of iron oxide enable the rapid separation of pollutant particles, surpassing the capabilities of centrifugation and filtration techniques. This magnetic feature also facilitates the development of efficient strategies for preventing the entry of pollutants into the environment. Moreover, iron oxide particles can be reused after magnetic separation and regeneration, making them a promising adsorbent for the removal of environmental pollutants [11].

**Figure 1:**
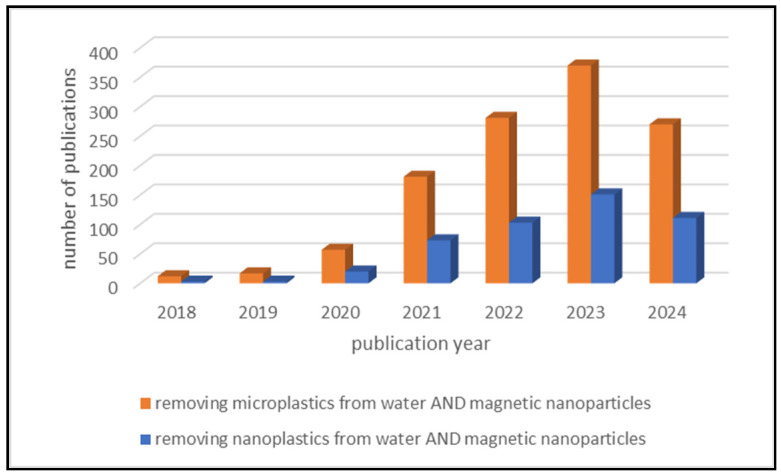
Research papers found using the keywords “removing microplastics from water AND magnetic nanoparticles” (orange color) and “removing nanoplastics from water AND magnetic nanoparticles” (blue color). The number of papers is shown according to the year of publication. The data were obtained by searching the ScienceDirect search engine on 16 April 2024.

Figure 1 uses columns to show how the number of publications related to the topics under investigation has increased over the years. Publications linked to the search term “removing microplastics from water AND magnetic nanoparticles” are shown with orange bars, while publications linked to “removing nanoplastics from water AND magnetic nanoparticles” are shown with blue bars. In both cases, it is clear that the number of publications has increased significantly in recent years, demonstrating the pressing global problem of water pollution by MPs and NPs and also showing how useful MNPs are in removing MPs and NPs from water.

In this review article, we aim to highlight the usefulness and effectiveness of using MNPs to remove MPs and NPs from water sources. Given the topic, we first wanted to highlight the problems associated with MPs and NPs and their impacts on the environment and human health. At the same time, we highlighted MNPs and their important properties that affect the efficiency of MPs’ and NPs’ removal from water. Based on existing studies, we focused on MNPs of different compositions and emphasized their advantages, limitations, and, of course, potential for further developments leading to a better efficiency of MNPs in the removal of MPs and NPs from water. Due to promising results obtained using these materials, a detailed literature review in this field is provided.

### Sources of Plastic in the Environment

Water serves as a significant carrier of MPs and NPs, as plastic particles can be released into the environment from various sources. Packaging materials, building materials, fishing gears, automotive parts, electronic utilities, and agro-industry components make up the majority of plastic waste, with household, medical waste, and sports equipment also contributing. The COVID-19 pandemic has led to the increased consumption of single-use plastics, such as personal protective equipment (PPE) kits, facemasks, and gloves, adding to the global solid waste fraction. Approximately 400 million tons of plastics are dumped into oceans alone. MPs and NPs are emerging as significant concerns, found in various commonly used products or formed through the fragmentation of larger plastic waste. Primary MPs are produced in large quantities for multiple domestic applications, including facial scrubs, toothpaste, detergents, cleaning agents, plastic powders, and synthetic clothing. NPs are released by paints, adhesives, electronics, and other sources. Secondary MPs/NPs result from the breakdown of macroplastics and account for a significant portion of plastic released into the environment. MPs and NPs can be found in terrestrial, freshwater, and marine ecosystems worldwide [12,13].

MPs and NPs in water pose a threat to aquatic organisms as they can be ingested, causing physical harm or toxic effects. Plastic particles can impact the food chain, as they can accumulate in organisms consumed by fish and other aquatic animals, gradually moving up the food chain [1,14].

Furthermore, there is concern that MPs and NPs could enter drinking water sources, as evidence of their presence has been found in water sources such as rivers, lakes, and groundwater. Although the current scientific understanding of the impact of MPs and NPs on human health is still limited, there are concerns regarding potential their long-term effects on the human body, including potential impacts on the digestive system, immune system, and hormonal balance [15,16,17,18,19].

Removing MPs and NPs from water is a complex challenge, as the particles can be extremely small and difficult to detect and effectively remove with traditional purification methods. The development and use of new technologies, such as the utilization of MNPs, as mentioned earlier, show promising potential for improving the removal of MPs and NPs from water sources [4,20].

Addressing the issue of MPs and NPs in water requires a comprehensive strategy that includes source control, the effective treatment of wastewater, promoting plastic recycling, and raising awareness of the issue. In this review, we first provide a concise introduction of the topic, including an overview of the main sources of MPs and NPs in water, the major methods for their detection, and their adverse effects on the environment and human health. The main aim of this review paper is to summarize the current advances in the techniques for the removal of MPs and NPs from aqueous media, with the primary focus being on methods utilizing iron-based MNPs, which offer multiple advantage due to their inexpensiveness, biocompatibility, and excellent magnetic properties. Continued research and collaborative efforts are essential to understand the extent of the problem, develop efficient removal methods, and mitigate the environmental and health risks associated with MPs and NPs in water.

## 2. Properties of MPs and NPs

The transport of MPs/NPs is influenced by factors like size, shape, and aging, in addition to soil properties. A study found that NPs (187 ± 22 nm) had a retention rate of 48.5 ± 7.8% in the column, while MPs (about 30 µm) had a higher retention rate of 94.4 ± 6.1%. This suggests that NPs are more likely to move downwards and potentially reach groundwater systems compared to MPs. Aggregation, whether between plastics or between plastics and other substances like organic matter and soil particles, increases aggregate size and affects transport. Additionally, fibrous MPs are often harder to transport compared to spherical or granular ones because of their thin, long shape, making them prone to directional loss and entanglement with soil particles. Notably, aging NP surfaces become more hydrophilic after oxidation, enhancing their mobility and ability to interact with both polar and non-polar pollutants, thereby increasing their contaminant-mobilizing capacity [21].

The intricate nature of MPs encompasses at least five dimensions that must be taken into account when analyzing these minuscule particles:

- A wide size range, ranging from 1 μm to 1 mm (and up to 5 mm for larger MPs).
- Diverse polymer types with varying chemical compositions, including both conventional and biopolymers with different structures and densities.
- Various shapes such as spheres, irregular particles, fibers, films, and foams.
- The incorporation of different additives (antioxidants, light stabilizers, plasticizers, flame retardants, pigments, etc.), weathering byproducts, and adsorbed contaminants (persistent organic pollutants, antibiotics, heavy metals, etc.).
- Different aging states (primary and secondary MPs), biofouling, surface charge, and hydrophobicity [2].

Analyzing the distribution of MP and NP particles provides crucial insights into their potential sources, transportation mechanisms, and interactions with living organisms [22]. The ecological significance of particle size cannot be overstated, as it stands as arguably the foremost factor in dictating the interaction of MPs with organisms, and their ultimate environmental destiny and size play pivotal roles in determining the quantity of plastics consumed. There are many classifications of MPs and NPs according to their size ranges, with one of the most common among them distinguishing between NPs (1–1000 nm; further divided into NPs of 1–100 nm and sub-MPs of 100–1000 nm), MPs (1–1000 µm), mesoplastics (1–10 mm), and macroplastics (>1 cm) [23]. In a study of plastic pollution in the north Atlantic ocean [24], PE was the most prevalent polymer, accounting for 90.3% of all plastics discovered. PP plastics made up 9.7%, while polymethylmethacrylate (PMMA) and PET were also detected, but each contributed less than 0.1%. The majority of both PE and PP MPs were in fragment form (81% and 68%, respectively), with only PE found in granulated form (0.28% of the total PE fraction). Fibers constituted 15% of PE and 20% of PP MPs. Additionally, 3.5% of the PE MPs were in film form, in contrast to 11.84% for PP. Given that both PE and PP are among the most commonly manufactured plastics and have densities lower than that of seawater, this discovery was not surprising. Other researchers sampling the North Atlantic have reported similar findings.

The concerns regarding the fate of the environment arise from the detection of plastic particles on a small scale. However, the constantly changing charges, shapes, sizes, and densities of these particles complicate the assessment of potential threats [3].

In recent years, research on NPs has primarily concentrated on refining methods for quantification, detection, and identification, with a particular emphasis on categorizing them according to their functional groups. Progress and innovation in this field may lead to the adoption of thermoanalytical techniques and spectroscopic analysis. The latest improvements in analytical methods for discerning the functional groups of NPs have leaned towards cost-effectiveness and standardization [25].

Table 1 below summarizes, based on article [26], the certain characteristics of some MPs in real samples from various environments. These properties were determined using various characterization methods.

## 3. Detection of MPs and NPs

**Figure 2:**
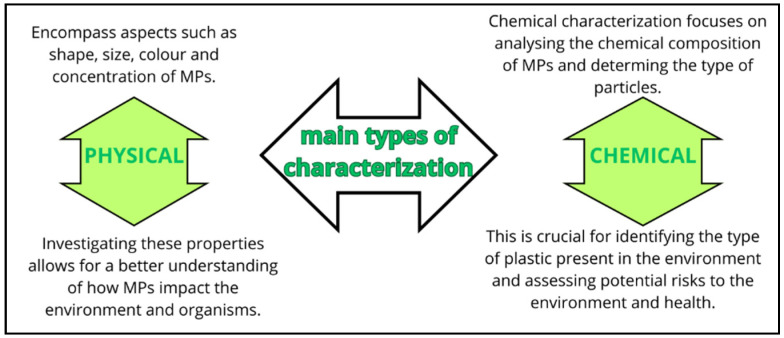
Two main types of characterization involving the analysis of MPs and NPs.

When discussing the detection of MPs and NPs, it is essential to acknowledge a series of steps and precautionary measures that must be taken into account for a comprehensive analysis. This process extends from the sampling and processing of samples to detection and identification. Although new methodologies are continuously evolving, the analysis of MPs and NPs typically involves two main types of characterizations, e.g., physical and chemical methods (Figure 2).

In conducting these analyses, precision, standardized methods, and appropriate measures to prevent sample contamination are of paramount importance. Researchers continuously strive to enhance and develop techniques for the improved detection and characterization of MPs and NPs in the environment [1,13,27].

The net sampling method is one of the fundamental techniques for collecting MPs from water in the natural environment, such as rivers, lakes, seas, and oceans. This method allows MPs to be collected from water as it passes through a net, which acts as a filter. Due to their smaller size, NPs cannot be sampled using nets [27,28]. The key factor in selecting the net is the mesh size, which determines the sizes of the MPs that are collected. The net is placed in the water where the sample is to be collected and can be attached to a frame or used with a special sampling device. Water passes through the net while MPs remain trapped in it. The sampling itself takes from approximately a few minutes to several hours, depending on the research objectives. The more time water passes through the net, the more MPs will accumulate on the net itself. Subsequently, the net is removed from the water, and gentle rinsing or the use of special solutions are employed to separate the MPs from the mesh. This is followed by drying and storing the MPs and further analysis of the sample, which assesses their size, shape, and color, with additional chemical analyses performed if needed [14,29]. The net sampling method is straightforward and relatively cost-effective, but it requires careful planning and execution to ensure the reliability of results. Moreover, it is crucial to consider factors such as water currents, which can affect the collection of MPs. This is a key technique for examining the presence of MPs in the natural environment and their potential impact on ecosystems [27].

Filtration methods are an effective way to collect MPs and NPs from water in the natural environment, as they enable the selective separation of particles, including plastic, from the water sample [3]. The choice of a filter with the appropriate pore size, tailored to the target size of MPs, is crucial in these methods. This ensures that MPs are captured while larger particles are prefiltered. Filtration can be conducted using a vacuum system or under pressure to expedite the process. After filtration, the filter is removed and cut into smaller fractions to facilitate further analysis. Fractions containing MPs and NPs are then transferred to the laboratory for subsequent analysis [27,30,31].

Sedimentation relies on the principle of gravity to allow MPs to separate and settle at the bottom of a container [14,27]. The process begins with the collection of a water sample from the target environment, ensuring that it represents the specific area of interest. The collected water sample is then transferred to a container, typically a sedimentation chamber or cylinder with a conical bottom, designed to facilitate the settling process. Once in the container, the sample is left undisturbed and allowed to stand for a specified period, often ranging from several hours to overnight. During this time, gravity causes the heavier MP particles to gradually sink to the bottom of the container. After the sedimentation period, the upper portion of the water, which is now relatively clear, is carefully decanted or siphoned off, leaving behind the sediment at the bottom of the container. The water can be removed until only a small amount remains above the sediment layer. The sediment layer contains the settled MP particles, as well as any other particulate matter that may have settled. The collected sediment is then carefully transferred to a separate container for analysis. Analytical techniques may include visual inspection, microscopic examination, and chemical analysis to identify and characterize the MPs found within the sediment [31,32,33].

Additional separation methods, including hydrophobic interactions, magnetic field extraction, and electrophoresis, can also be employed for the purpose of separation. However, further progress is required to ensure their efficient and dependable utilization in the analysis of MPs and NPs [34].

**Figure 3:**
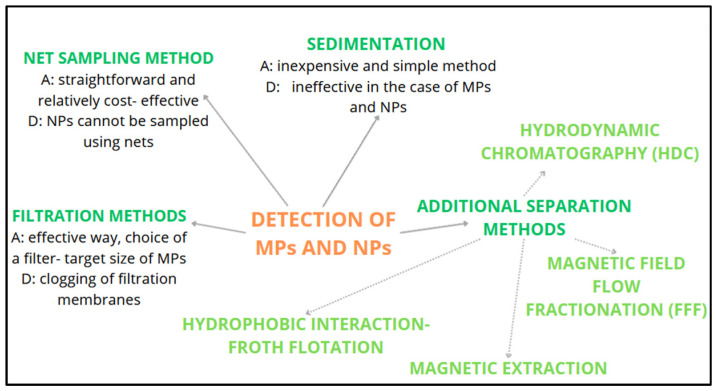
Presentation of various methods for the detection of MPs and NPs, pointing out their main advantages (A) and disadvantages (D). Some of them are already well researched, while others are just being developed.

Froth flotation relies on hydrophobic interactions, where plastic particles attach themselves to the surfaces of bubbles and are transported to the air–liquid interface. However, conventional froth flotation often yields low particle recovery due to challenges in controlling bubble size [35]. Magnetic extraction is another method under investigation for the separation of plastic particles. In this approach, MNPs are rendered hydrophobic through silanization, enabling them to bind to plastics and be separated from the matrix [36]. Modified separation techniques, such as magnetic field flow fractionation (FFF), offer potential for separating plastic particles of different sizes [34]. FFF is an active chromatographic technique widely used for the separation of MPs. In FFF, an external force, such as gravity, thermal gradients [37], centrifugation [38], magnetism [39], or electricity [40], is applied through an asymmetrical flow across a semi-permeable membrane [41]. This force acts perpendicular to the flow direction, allowing for the separation of dispersed particles based on their differential mobility [42]. Hydrodynamic chromatography (HDC) is another method under investigation for separation purposes. HDC is considered to be a passive chromatographic technique that relies on hydrodynamic and surface forces to separate particles within a liquid medium. HDC is known for its ease of use and rapidity, offering analytical repeatability; however, it exhibits a lower selectivity in terms of pore size resolution when compared to FFF [26]. An overview of the commonly used detection methods, including the references, is provided in Figure 3.

If we want to obtain information about the shape, size, and concentration of MP particles, various techniques can be used. Among the most commonly used are microscopic techniques, including Optical Microscopy, Scanning Electron Microscopy (SEM), Transmission Electron Microscopy (TEM), and Atomic Force Microscopy (AFM). Optical microscopy is primarily used to obtain information about the size, shape, and concentration of MP particles. Stereomicroscopic imaging provides a better discrimination compared to visual sorting, but it is generally unsuitable for particles smaller than 100 µm [43]. Meanwhile, fluorescent imaging is more selective and allows for the identification of transparent and white plastic particles [44]. Using optical microscopy, plastic particles can be categorized into groups based on their shape, size, and color. For more precise identification, other microscopy techniques are also available. A higher resolution compared to optical microscopy is achieved by SEM, which was introduced to study the morphology of particles obtained from various samples [45,46,47]. When SEM is used in combination with energy dispersive X-ray spectroscopy, we not only obtain information about the size and shape of particles, but also their elemental composition or chemical identity. In the case of MPs, this analysis is complex, as MPs are non-conductive and the sample preparation itself can be very time-consuming [48,49]. In general, TEM is employed for the characterization of nanomaterials due to its ability to achieve a spatial resolution down to the atomic scale. However, when it comes to the analysis of MPs and NPs, TEM has a limited applicability. Firstly, NPs exhibit an amorphous structure, are non-conductive, and necessitate extensive metallic staining for reasonable contrast, rendering TEM ineffective for visualizing NPs. Moreover, the high-energy electron beam can potentially damage the particles. TEM requires complex instrumentation, is costly, and lacks user-friendliness, thereby restricting its use in resource-constrained settings. Nevertheless, TEM is still utilized in specific contexts, such as the examination of the impact of MPs on model systems. For example, Sun et al. [50] employed TEM to investigate the toxic effects of PS MPs and NPs on the marine bacterium *Halomonas alkaliphila*, and Song et al. [51] utilized TEM to assess the potential effects of MPs on microalgae. The AFM technique offers significant advantages when it comes to characterizing nanoscale particles, and it is not restricted to conductive samples. AFM is capable of providing high-resolution three-dimensional images, with resolutions down to a few nanometers (up to 0.3 nm). It involves straightforward sample preparation, making it suitable for investigating the surfaces of non-conductive materials like MPs and NPs, all while avoiding sample damage from radiation. However, there are certain limitations associated with AFM. For instance, it is susceptible to external contamination, and the AFM tip can potentially damage the sample by releasing fragments to the tip, leading to inaccuracies in the image of the sample. While AFM instrumentation is more complex compared to traditional optical microscopes, AFM imaging has the potential to find numerous applications in the analysis of MPs in various matrices [52].

Another important group of methods for the identification of MPs and NPs are vibrational spectroscopic techniques, among which Infrared and Raman Spectroscopy are included. Fourier transform infrared spectroscopy (FTIR) is a widely employed technique for characterizing substances based on their vibrational frequencies, specifically, the bonds present in a molecule. The choice of mode in FTIR analysis depends on the nature of the sample. The transmission mode is applicable to thin samples that allow for the infrared (IR) beam to pass through. The diffuse reflectance mode is suitable for fine powder samples (<10 µm), while thick or strong-IR-absorbent samples often require the attenuated total reflection (ATR) mode. In cases involving large, flat, and reflective surfaces, the true specular reflectance/reflection-adsorption mode is preferred [13,43]. FTIR is utilized for identifying and characterizing plastic polymers, as well as for obtaining information about the physicochemical weathering of plastic particles. However, there are associated challenges. Surface-contact analysis, such as ATR-FTIR, can potentially damage small and fragile plastic particles due to sample–tip adhesion or electrostatic interactions. Additionally, sample drying is necessary before IR spectroscopy, since water strongly absorbs IR radiation. Traditional FTIR cannot provide spectroscopic information at the single nano or small MP particle level. The smallest size that can be studied at the single-particle level is approximately 250 µm. To address these limitations, the combination of IR spectroscopy with an IR microscope, collectively known as micro-FTIR (µ-FTIR), has become a highly utilized imaging technique in MP analysis. Unlike traditional IR spectroscopy, micro-FTIR offers both the morphological and chemical identification of MP particles larger than 10 µm, with a spatial resolution of approximately 5 µm. However, it is worth noting that micro-FTIR imaging requires longer acquisition times, making it challenging to access the entire filter area [53]. Raman spectroscopy is a highly favored technique for identifying MP powders or particles (approximately 500 µm) within different environmental samples. In recent years, µ-Raman imaging has gained significant popularity for particle analysis, because it can provide both spatial and chemical information for particles as small as 10 µm, with a remarkable spatial resolution of around 1 µm [54,55]. Raman spectroscopy boasts several advantages over FTIR. It typically allows for non-destructive analysis and is versatile in terms of sample thickness or whether the sample is in a solution, gas, or film, on a surface, in solid form, or is made up of single crystals. Moreover, Raman analysis can be conducted at various temperatures. However, a notable challenge in obtaining high-quality Raman spectra is the presence of fluorescence. To address this issue, it is recommended to include a sample purification step before performing Raman spectroscopy. Additionally, the use of baseline removal algorithms or more efficient detectors can effectively mitigate fluorescence-related problems [56].

Additionally, there are thermo-analytical techniques, including Differential Scanning Calorimetry (DSC), Thermogravimetry (TGA), and Gas Chromatography-Mass Spectrometry (GC-MS), as well as other techniques such as Dynamic Light Scattering (DLS), Nanoparticle Tracking Analysis (NTA), and others [1,12,13,14]. MP particles extracted from a processed sample can be distinguished through a range of thermo-analytical methods. These techniques are valuable because plastic polymers exhibit varying degrees of thermal stability. Thermo-analytical techniques enable the detection of alterations in the physical and chemical characteristics of polymers following degradation. Consequently, they facilitate the identification of MPs and NPs present in diverse matrices [57]. DSC is a technique that assesses temperature changes and associated heat flux during transitions in a sample. It provides insights into various characteristics of polymeric materials, including melting enthalpies, glass transitions, and crystallization kinetics. DSC is particularly effective in identifying primary MPs with well-defined properties, as it relies on reference materials for comparison. However, when dealing with a mixture of MPs that share similar melting points, DSC may lack specificity in its analysis [52].

TGA is a method that quantifies the mass loss from a sample at specific temperatures and generates thermogravimetric profiles, illustrating mass changes concerning temperature. It is commonly employed when studying polymeric materials, which frequently undergo degradation accompanied by enthalpy alterations [52]. Combining TGA with DSC allows for the measurement of enthalpy changes, making this combination advantageous for MP analysis [58]. While this approach is effective for polyethylene (PE) and PP, it may not be suitable for identifying other polymers like PVC, polyamide (PA), polyesters (PES), PET, and polyurethane (PU), due to the potential overlap of phase transition signals [57]. In recent years, GC–MS has found applications in the chemical identification of MPs through mass fragmentation patterns. Additionally, GC–MS serves as a valuable tool for examining adsorbed organic substances and volatile plastic additives present on MPs. Moreover, the potential of GC–MS can be further harnessed through its integration with other techniques such as pyrolysis and thermal desorption, enhancing the characterization of MPs [59,60,61]. In recent years, there has been a growing interest in exploring emerging techniques for the identification and quantification of MPs and NPs, particularly in environmental samples. Here, we provide a concise overview of these methods. DLS is a commonly employed method for examining the size distribution of MPs and NPs, such as polymeric colloidal MPs and NPs in solution phases. It is effective within a size range from 1 nm to 10 mm [26,62,63]. For instance, Gigault et al. utilized DLS with a photo-detector to investigate the photo-degradation of marine MPs [64]. NTA represents another potential method for examining the distribution of NPs in environmental samples. NTA has the capacity to detect individual particles as small as 10 nm and provides information about individual particle sizes, as opposed to offering average size data, as seen in DLS. It is worth noting that the accuracy of NTA can be limited by Brownian motion, so particles should not be too close or too polydisperse.

## 4. Impact on the Environment and Human Healthy

More than 80% of MPs are produced on land, with less than 20% coming from the ocean. Because MPs are particularly light and indestructible and can float, they can travel far around the world [15,65]. Most of the plastics that pollute the marine environment come from land-based sources, fisheries and other aquaculture, and coastal tourism. It is estimated that there are over 800 million tons of land-based plastic in the ocean [66]. Because MPs and NPs are incredibly small, they cannot be filtered in wastewater treatment, thus, such plastic particles end up in rivers and oceans, as well as freshwater supplies. In addition, MPs and NPs are found in the soil and also enter rivers and oceans through natural erosion [67].

MPs and NPs are produced from primary and secondary sources [68,69]. Primary sources are the intentionally produced MPs and NPs for consumers and industry, e.g., as exfoliants in detergents and cosmetics and as drug delivery particles in medicines, as well as in industrial air blasting. Macroplastic products, which break down into micrometres and smaller particles, serve as secondary sources of MPs and NPs; they occur both on land and in aquatic environments [15,70].

Plastics can undergo degradation in the environment through six distinct processes: thermal degradation, thermo-oxidative degradation, biodegradation, photodegradation, mechanical degradation, and hydrolysis [16,68]. Thermal degradation takes place when plastics are exposed to elevated temperatures generated in industrial processes. Consequently, this form of degradation does not occur spontaneously in the natural environment [16,71,72]. On the other hand, thermo-oxidative degradation pertains to the gradual oxidative breakdown of plastic transpiring at comparatively lower temperatures. Plastics can also undergo decomposition due to microbial activity, including bacteria, leading to biodegradation [16,73,74]. Sunlight radiation provides another mechanism for breaking down plastics into smaller particles, a process known as photodegradation. Additionally, the mechanical wear and tear of plastics in the environment can result in the creation of even smaller particles. Plastics encountered in marine settings typically undergo hydrolysis, a form of chemical degradation caused by water [75,76]. NPs are regarded as highly dynamic within the environment, primarily due to their substantial surface area-to-volume ratio. This extensive surface area allows them to interact with their surroundings, influencing their bioavailability and mobility [74,77]. These particles have the ability to aggregate with each other or with other nano-sized materials nearby, resulting in changes to their surface area and chemical properties. When they come into contact with naturally occurring organic materials on a larger molecular scale, they develop a bimolecular corona, creating a new surface layer around the particle. NPs or the altered particles can also interact with living tissues, both extracellular and intracellular, influencing their bioavailability, transportation, and toxicity. Additionally, chemical reactions like oxidation and reduction can impact these characteristics. These transformations can occur over an extended period, spanning from several months to a few years.

The presence of MPs and NPs in various ecosystems, including in water, air, and on land, has garnered significant attention from the scientific community. Their persistent existence can lead to serious detrimental impacts on the environment and human health, both directly and indirectly [12,75].

**Figure 4:**
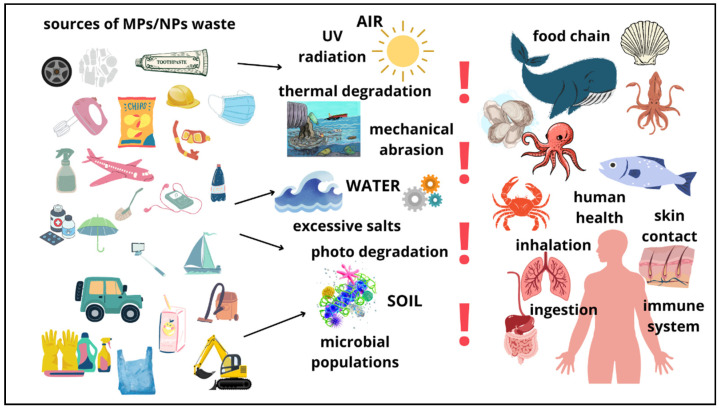
Schematic representation on the harmful effects of MPs and NPs on human health.

MPs and NPs, along with associated chemical additives, heavy metals, and organic pollutants, collectively constitute a complex pollutant system in urban waters. These pollutants have exhibited ecotoxicological effects on aquatic organisms and even humans [78,79]. Compared to MPs, NPs have an exceptionally higher proportion of adsorbed additives/molecules/contaminants on their surface, leading to a significantly greater surface reactivity and biological availability. More importantly, the size of NPs is close to that of natural proteins, making it easier for NPs to traverse biological membranes through passive diffusion and endocytic pathways [21,80]. At the water’s surface, MPs and NPs can serve as environments for viruses and bacteria because of their low density, easy suspension, and pronounced hydrophobic properties. As microbial populations accumulate on MPs and NPs, they give rise to microbial films that subsequently migrate to the deep ocean. Numerous studies have examined the ecological toxicity of MPs and NPs, which are commonly ingested by marine organisms [1,81]. Figure 4 shows the different sources of MPs/NPs and their harmful effects on human health.

Bivalves such as oysters, clams, shellfish, and mussels have been employed as research models due to their status as a primary human food source, with the food they consume directly entering their digestive system [81]. MPs and NPs can accumulate through the food chain, posing a risk to the health of animals. Large quantities of MPs have been found in the digestive systems of stranded or deceased seabirds on beaches [75,82]. Accumulated MPs and NPs can damage the digestive system at the tissue, cellular, molecular, and organic levels, leading to swelling of the affected tissues and triggering an allergic immune response. This plastic can block and harm the digestive tract of birds, ultimately leading to their demise [83,84,85]. Previous studies have also shown that the ingestion of MPs and NPs can hinder the reproduction of migratory seabirds and affect the reproduction of mammals [86]. MP and NP fingerprints have been discovered in the intestines of fish, deep-sea species, large mammals, and benthic invertebrates at various trophic levels. It has been found that surface water contaminated with MPs can impede the respiration and photosynthesis of marine plankton [87,88]. NPs and MPs exert varying effects on plants, such as plankton and macrophytes, within urban water systems. According to reports, almost 90% of studies examining the impact of NPs on phytoplankton indicated that NPs had toxic effects, whereas approximately 90% of relevant studies suggested that MPs (1–10 μm) displayed no toxicity [89]. In general, MPs primarily affect plants by obstructing their nutrient transport channels. In contrast, NPs, which can be absorbed and traverse plants, exhibit a more intricate phytotoxicity. Furthermore, NPs can internalize within plant tissues, leading to damage through the regulation of cell membranes and endomolecules, as well as reactive oxygen species (ROS)-induced genotoxicity and cytotoxicity [90,91]. MPs and NPs are introduced into the atmosphere through various sources, including synthetic textiles (such as clothing, furnishings, and carpeting), the wear and tear of materials, and the re-suspension of MPs and NPs from waste, landfills, and emissions [92,93]. Given their small size and low density, plastic particles can readily become airborne, serving as effective carriers of diverse organic pollutants in the atmosphere. These airborne MPs and NPs can be transported over substantial distances, eventually undergoing wet or dry deposition onto oceans, freshwater systems, and land. When comparing the migration processes of MPs and NPs through the air to their aquatic transportation counterparts, airborne transport faces fewer topographic constraints. Airborne MNPs can be easily dispersed in multiple directions and exhibit a prolonged persistence [81,94].

MPs and NPs, resulting from plastic fragmentation caused by temperature and photo-oxidation, contaminate soil by infiltrating soil layers and originate from various sources, including sludge recycling, wastewater irrigation, fertilizers, landfill disposal, biosolids, and others.

Notably, the quantity of MPs and NPs transferred to soil has recently surpassed the amount found in the ocean. The presence of MPs and NPs alters the physicochemical properties of the soil, affecting factors such as porosity, soil structure, water retention capacity, soil bulk density, and more. Furthermore, MPs and NPs influence the physiological activities of soil-dwelling organisms, resulting in growth inhibition and harm to intestinal and immune systems, among other adverse effects. Researchers have discovered that nematodes readily ingest MPs and NPs, accumulating them first in the middle and subsequently in other parts of the gut. In addition, MPs and NPs impact plant growth and germination, disrupting the ability of plant roots to absorb water and nutrients [81,92]. To gain a more comprehensive understanding of the impact of MP and NP pollution, it is crucial to consider certain key parameters. MPs and NPs with smaller sizes tend to have a more pronounced biological impact compared to larger ones. Additionally, particle shape is a significant factor, with irregular particles appearing to cause greater physical effects when compared to round particles. Concentration is also of substantial importance in toxicological studies. Both in vivo and in vitro research often employ higher concentrations of MPs and NPs than those found in the environment. Nevertheless, a thorough investigation is needed to precisely assess the distribution of MPs and NPs across all components of the environment and their detrimental effects on living organisms in order to accurately estimate the impacts of MP and NP pollution [12,94].

The fact is that plastic has become an integral part of human needs and is used worldwide. Due to its fragmented forms (MPs and NPs), the question of the origin of plastics arises in many respects. As a result, MPs and NPs have become a significant threat to the environment, as these materials can easily become contaminated with various other hazardous substances, creating harmful conditions for living organisms. As plastic waste continues to increase, the presence of MPs and NPs in the food chain also poses a threat to human health. Due to their broad biological benefits and ubiquity in the aquatic and terrestrial environment, it is very likely that MPs and NPs are present in numerous food products [19,70,75]. Human exposure to MPs and NPs can occur through various means, including the consumption of contaminated food and water, inhalation of indoor and outdoor air, and contact with fabrics, personal care products, or indoor dust. These minute particles can enter the human body through the digestive system, respiratory system, and skin contact [16,95,96]. Inhaled MPs come from urban dust and contain synthetic textiles and rubber [95]. Nowadays, it is very difficult to avoid ingesting MPs, as they are widespread in the food chain and water supply [18,70]. If the skin membrane is impermeable to MPs or NPs, it can penetrate through wounds, sweat glands, or hair follicles [16,79,97]. All three routes of entry contribute to the total amount of MPs and NPs in the human body, but particles in seafood and the environment pose the highest risk of absolute exposure. This is due to the prolonged degradation of polymers, leaching of chemical polymer additives, residual monomers, and exposure to pollutants and pathogenic microorganisms active in these environments [78,98]. Of all three routes by which MPs and NPs enter the human body, studies have shown that most particles are ingested directly, which has attracted considerable scientific and public attention to this area [17,68,78]. The studies carried out indicate that MPs are mainly ingested through food and drink, which has been confirmed by analyses of human stool samples [99,100]. The numbers clearly show that humans ingest exceptionally large quantities of MPs and NPs. However, what exactly happens to MPs and NPs when they enter the gastrointestinal tract is not yet known [17]. Most studies used models with PS nanoparticles and neglected other polymers found in the environment (PP, PE, and PET) [70]. However, the most concerning fact is that the absorption of MPs and NPs gives rise to numerous health concerns related to particle toxicity, chemical toxicity, and the introduction of pathogens and various parasites [16,75]. The second most probable pathway of human exposure to NPs is through inhalation. Airborne plastic particles, mainly derived from synthetic textiles, are present in indoor environments, resulting in inadvertent inhalation or occupational exposure. In outdoor settings, exposure may occur through the inhalation of contaminated aerosols generated by ocean waves or airborne fertilizer particles emanating from dried wastewater treatments [78,96,101]. The absorption of plastic particles, particularly MPs and NPs, raises various health concerns, including particle toxicity, chemical toxicity, and the introduction of pathogens and parasite vectors. Particles within this size range have the potential to penetrate deep into the lungs, remaining on the alveolar surface or translocating to other parts of the body. The inhalation-induced absorption of plastic particles may result in lung damage [101,102,103]. Health and beauty products represent a significant source of NPs, especially in body and facial scrubs applied topically to the skin [104]. Another crucial exposure route is through nanocarriers used for drug delivery via dermal application. While conclusive data on the effects of nanocarriers are lacking, their small particle size and compromised skin conditions are critical factors influencing skin penetration [97]. The stratum corneum is the outermost layer of the epidermis and serves as a protective barrier against injuries, chemicals, and microbial influences [105]. As MPs and NPs are hydrophobic, the absorption of contaminated water through the stratum corneum is not expected. If the skin is damaged by UV rays or small tears, MPs and NPs can penetrate the skin barrier. Researchers have shown that medical devices implanted in the human body, such as PE joint spacers and cosmetic and dental implants, facilitate the production of MPs and NPs and their subsequent translocation to other parts of the body [16,106]. It is important to emphasize that there is still a lack of data on the direct effects of MPs and NPs on human health and that further research should be conducted in the future to comprehensively assess the effects of MPs and NPs on the human body and human healthy.

## 5. The Techniques for Removing MPs and NPs from Water

The development of methods for removing MPs and NPs from aquatic environments has been significantly increasing in recent years, primarily due to the risk of NPs’ presence in drinking water and their potential health hazards for humans [107,108]. The task of treating drinking water involves the removal of physical, chemical, and biological pollutants such as solid particles, heavy metals, and microorganisms, as well as the removal of NPs/MPs from water. Several different techniques and methods have been employed for the removal of MPs from drinking water [3]. Traditional methods for drinking water treatment include processes such as coagulation, sedimentation, and sand filtration. These procedures hold promise for the removal of MP particles from water too. However, to improve the removal of MPs and NPs from water, it is necessary to thoroughly study the size distribution of MP particles and their interaction with other pollutants [109].

In Table 2, some traditional methods for removing MPs and NPs from water, including filtration, ultrafiltration, centrifugation, flocculation, photocatalytic degradation, and membrane bioreactor filtration, are summarized [3].

**Figure 5:**
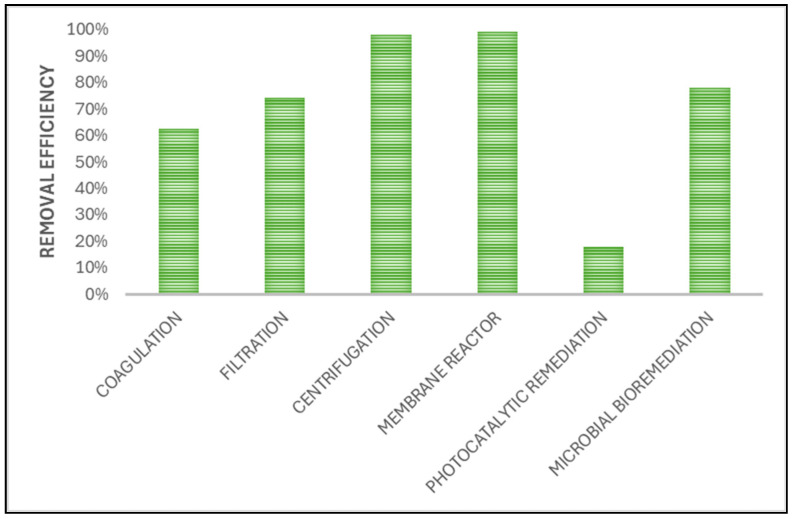
Efficiency of NP removal with various methods.

Below, in Figure 5, a graphical representation of the efficiency of NP removal with various methods is presented (summarized by [3]).

### 5.1. Membrane Filtration

**Figure 6:**
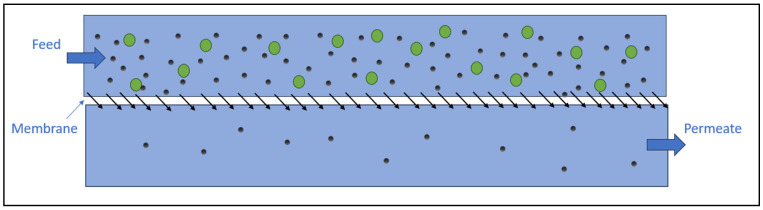
Schematic representation of membrane filtration.

There are several types of membrane filtration used to remove MPs and NPs from water, which are distinguished by the size of the membrane. These include ultrafiltration, nanofiltration, and reverse osmosis (RO) [3]. The filtration of MPs using a membrane is the most basic method for separating MPs from liquids. The advantage of this method is the stability of the effluent quality and its easy operation [110]. The particles being separated come from various substances and have different forms. This is the reason why we utilize membranes with different pore sizes, through which water passes under pressure; the pressure that allows for the most efficient removal of MPs is between 100 and 400 kPa. Membranes can be made of polycarbonate, cellulose acetate, polytetrafluoroethylene, and many other materials. The main problem, which also reduces the efficiency of this method, is membrane fouling. Shen and colleagues [109] found that MP particles are negatively charged. Therefore, it makes sense to choose a membrane material with a negatively charged surface, as it will repel negatively charged particles, resulting in less fouling. The lifespan of membranes greatly increases when in-line membrane flushing is used. Membrane filtration can achieve an unsatisfactory efficiency in removing MPs and NPs due to the incorrect choice of pore size in the membrane. If the pores of the membrane are slightly larger, some plastic particles remain unremoved. In Figure 6, a schematic representation of membrane filtration is shown.

#### 5.1.1. Ultrafiltration

Ultrafiltration is an important method for removing NP particles from water. Due to the action of hydrostatic force on membranes with nano-sized pores, particles are separated from the carrier medium [3]. Enfrin and colleagues [111] achieved the removal of more than 25% of previously present MP and NP particles using ultrafiltration, with an operating time of 48 h. A limitation of filtration in removing particles from water is the potential leakage of particles smaller than the pore size of the membrane into the water, which can have a significant negative impact on aquatic life [112].

Ultrafiltration, combined with two or more processes, can be a promising method for removing NP particles from drinking water. Arenas and others [113] reported that, by filtering drinking water with sand and granular activated carbon, they removed 88.1% of NP particles without coagulation. Meanwhile, when using coagulation filtration, they removed more than 99% of NP particles from water.

#### 5.1.2. Reverse Osmosis

RO is one of the most recognizable and important processes in the water treatment industry. In addition to removing nitrates from water, RO also allows for the removal of pesticides or other organic materials that may be present in the water. Water treatment with RO produces water with low levels of organic impurities, but it requires a significant amount of energy for its operation [114]. Commercially used RO membranes are typically made of two polymers (cellulose acetate and PA). The use of these materials provides the best current selectivity and permeability [115]. RO is commonly employed to achieve high efficiencies in removing MPs from water.

Ziajahromi and colleagues [116] investigated the removal of MP particles larger than 25 μm from water using RO. They found that RO effectively stopped various types of MP particles, including PE, PS, PET, and PP. The stopped particles were mostly in the size range from 100 μm to 190 μm. The achieved efficiency of the process was 90%. The liquid that passed through the membrane contained only PET. The researchers attributed this to the size of the membrane pores and the membrane material.

### 5.2. Centrifugation

The key variables for optimal centrifugation results are time and speed, which vary depending on the size of the targeted removed NPs. The primary idea of the methodology is to eliminate separated particles from the solution to prevent their re-entry into the removal process [3]. Nguyen and colleagues [34] investigated various centrifugation times and speeds for removing NPs from water. A study by Lya and others [117] demonstrated that increasing the speed enhanced the efficiency of removing MPs and NPs from water, achieving a successful removal rate of 50%. Bannick and co-workers [118], employing high-speed centrifugation (10.000 rpm), achieved an almost 90% successful removal of MP particles from water.

### 5.3. Flocculation

**Figure 7:**
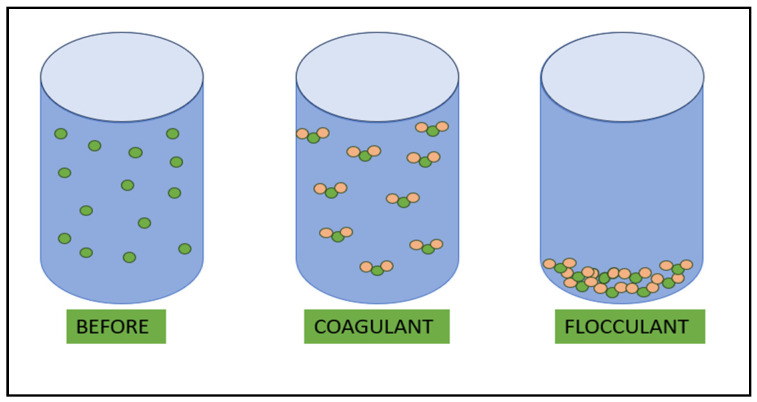
Flocculation scheme.

Flocculation (Figure 7) is suitable for the removal of certain multivalent metal cations, such as magnesium and aluminum cations, in the treatment of water. At high pH values, lime is commonly used in water treatment, acting as a precipitant to remove heavy metal ions, phosphates, and bacteria, or for the coagulation of suspended and colloidal material. With lime or NaOH acting as a pH regulator, both calcium carbonate (CaCO_3_) and magnesium hydroxide (Mg(OH)_2_) form at pH > 10.5. Carbonate ions (CO₃^2^⁻) are negatively charged and capture larger particles during their formation. Mg(OH)₂, due to its surface properties, can trap negatively charged colloidal particles, including CaCO₃, because of its large adsorptive surface area [119].

Researchers have found that the described method does not achieve a high efficiency in removing small NP particles from water [120]. Batool and Valiyaveettil [121] reported an efficiency of about 77–87% in removing NPs from water using flocculation with aluminum sulfate and some other salts. However, the flocculation method does not reach the same level of efficiency as ultrafiltration and centrifugation [122].

### 5.4. Photocatalytic Degradation

The method of photocatalytic degradation is employed for the breakdown of plastic material into low-molecular-weight components. In this method, a photocatalyst, such as titanium oxide, is utilized along with solar radiation to convert the plastic material into significant intermediate products [123]. The method is economical, as it harnesses renewable energy. In the initial stage of degradation (oxidation), the compounds formed are initially unstable. The second stage of degradation occurs in the environment, but the previously formed unstable compounds do not negatively impact human health and pose no risk to the aquatic environment [3].

Castelvetro and colleagues [124] investigated the photocatalytic degradation of NPs and confirmed the potential of this method in removing them from water. The degradation of NPs is believed to be associated with a semiconductor material exhibiting photocatalytic activity. Studies have demonstrated that this method can remove NPs such as PS from water [125,126].

### 5.5. Bioreactors

Among bioreactors, membrane bioreactors are utilized for the removal of NPs. These reactors operate with biological catalysts such as enzymes and bacteria, employing a vapor separation process through a membrane system [127]. This technology has been increasingly used recently, particularly in the removal of NPs from drinking water and wastewater [3]. The key process of this technique must have a high efficiency with an optimal retention time in hydraulic separation. Aeration is the main source for mechanical and enzymatic action, while chemical degradation can be electrically stimulated for NP removal based on the membrane [107]. The main advantage of this process, in addition to improving the removal efficiency, is the quality of the effluent, which undergoes a minimal number of treatment stages.

The use of bioreactors in water treatment processes has expanded significantly worldwide. In China, there is a high-tech hydroelectric plant named Beijing Wenyu, equipped with numerous membrane bioreactors [128]. In Europe, specifically in Sweden, a water treatment process based on bioreactors has been implemented, achieving a high efficiency by removing up to 99% of NPs from water [129].

### 5.6. Improved Adsorption

Adsorption can be enhanced by employing a suitable adsorbent for NPs in aqueous media. The most extensively researched area of this method is the removal of PS NPs. The mechanism of NP adsorption is associated with a reduction in surface charge and agglomeration [3].

Ramirez Arenas and colleagues [113], in their investigation on removing PS NPs from drinking water, using granular activated carbon as an adsorbent, finding that the surfaces of granular activated carbon and NPs became charged. The study also revealed that the adsorption capacity of NPs depends on the concentration of PS, reaching a maximum of 6.33 ± 0.20 mg/g. By combining coagulation and filtration processes with granular activated carbon as an adsorbent, the previously described method was improved to an efficiency of 99.2%. Zhou and others achieved a 75% effective removal of PS NPs from water using adsorption, where a Cu-Ni carbon material served as the adsorbent. They argued that PS adsorption with coagulation using magnetic materials is possible due to electrostatic attraction, complexity, and van der Waals interactions [130].

### 5.7. Utilizing Nanomaterials

**Figure 8:**
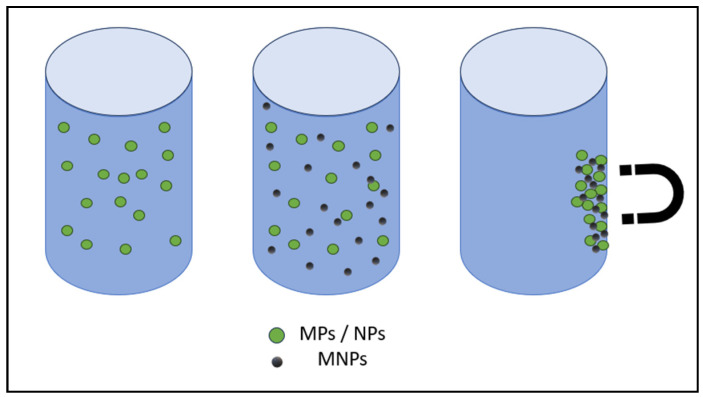
Removing NPs/MPs from wastewater by MNPs.

Currently, the most promising technique for removing NPs from wastewater and drinking water involves the use of various nanomaterials such as chitosan, metallic, polymeric, zeolitic, bimetallic, carbonate, magnetic, ferritic, and metallic oxides [3]. The magnetic separation of nanoparticles is faster than centrifugation and filtration. MNPs are suitable for use because, in addition to typical nanomaterial properties such as a small particle size and large specific surface area, they also exhibit superparamagnetic characteristics, making them suitable for various analytical applications [131]. In Figure 8, a schematic diagram of removing NPs/MPs from wastewater by MNPs is shown.

Shi and colleagues [132] investigated the removal of PS from water using biochar modified with cetyltrimethylammonium bromide. Due to the strong electrostatic charge among nanoparticles, PS retained a high stability in alkaline conditions. The removal efficiency was 67.4% at pH 11. With an increase in NaCl concentration, the inhibitory effects of NaCl on the removal efficiency of PS decreased.

## 6. MNPs (Properties, Synthesis Methods, Functionalization)

Nanoscience and nanotechnology are disciplines that deal with particles in the nanometer range and emphasize that these particles differ significantly in their properties from coarse-grained materials. This distinction is the main reason for the revolutionary developments of recent years. Nowadays, nanotechnology is making rapid progress in various directions, especially in the sectors with the highest financial investment, such as information technology, biopharmaceuticals, and biomedical engineering. One of the materials that has become particularly important in recent years is undoubtedly MNPs. They are the subject of research interest and are technically and biomedically useful due to their distinct specific magnetic, magnetoresistive, and magneto-optical properties, which are not observed in coarse-grained materials. MNPs exhibit extraordinary properties and associated phenomena due to their small dimensions (finite size effects) and, consequently, to the surface efficiency of these particles (influence of particle shape and surface) [133,134,135]. The chemical materials that give nanoparticles magnetic properties include iron, cobalt, or nickel, their oxides, and various other elements that combine several metals, such as copper, zinc, manganese, strontium, and barium [136]. Among these elements, superparamagnetic iron oxide nanoparticles (SPIONs), especially magnetite (Fe_3_O_4_), maghemite (γ-Fe_2_O_3_), and metal-substituted ferrites (MFe_2_O_4_ (M = Co, Cu, Ni, Mn, Mg, etc.), have attracted overwhelming attention in the last decade due to their biocompatibility, chemical stability, cost-effectiveness, and unique magnetic properties [137,138]. Of all morphologies, spherical nanoparticles have been the most intensively researched. The most common applications of MNPs (e.g., magnetic resonance imaging (MRI), hyperthermia, or tissue regeneration) are traditionally based on this geometry [138,139,140]. Furthermore, in MNP systems with multiple components, the magnetic properties of the nanoparticles can be adjusted by their composition, which influences the saturation magnetization and the coercivity of the resulting multicomponent systems.

The properties of magnetic multicomponent nanoparticles are highly dependent on their dimensions, composition, size, and structures, leading to extensive efforts to precisely control these parameters. Various methods such as co-precipitation, microemulsion, sol–gel, and hydrothermal/solvothermal techniques have been used to synthesize nanoparticles with magnetic properties. In particular, the synthesis of MNPs in the organic phase offers significant advantages over conventional hydrolytic methods, as it allows for better control over uniformity and structure [134,141]. In addition, magnetic nanomaterials often tend to agglomerate due to their large surface area, surface energy, and magnetic dipolar interactions, which requires stabilization by an organic or inorganic layer. This stabilization layer also helps to impart multifunctionality to the nanoparticles [142,143,144].

**Figure 9:**
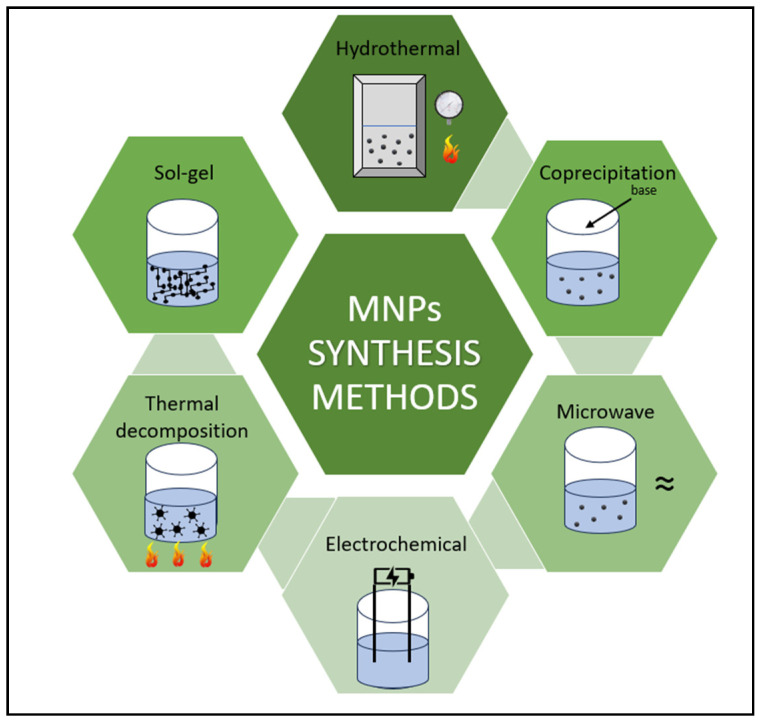
Synthesis methods for the MNPs preparation.

Various methods are available for the synthesis of MNPs (Figure 9), each of which has its own advantages and special features [145]. There are several factors to consider when synthesizing MNPs, such as particle size and shape, chemical purity, particle stabilization, property tailoring, etc. The synthesis methods can influence the size, shape, and distribution of the particles, which, in turn, affect their final properties [134]. To achieve the desired magnetic properties, pure synthesis is crucial. Since the particles tend to aggregate, the stabilization of the particles by a layer of organic or inorganic materials is essential. By changing the composition or by introducing surface coatings (e.g., polymers, biological molecules), the properties of nanoparticles can be adapted to the respective applications [133,140,145].

Co-precipitation is one of the most widely used methods [146], along with thermal decomposition, for the synthesis of MNPs, both in industry and in the laboratory. In this technique, aqueous solutions containing Fe^2+^ and Fe^3+^ ions are precipitated using alkaline solutions in an inert atmosphere, usually at room temperature or higher.

The typical bases used in coprecipitation are NaOH, N(CH_3_)_4_OH, and NH_4_OH. In addition, surfactants, polymers, or other organic compounds are usually added. These substances act as capping ligands that facilitate the achievement of the desired particle size and promote the dispersion of the particles by creating steric barriers between them [133,134,147].

A microemulsion represents a thermodynamically stable and isotropic dispersion comprising two normally immiscible phases: water and oil. This phenomenon occurs in the presence of a surfactant that aids in forming micelles, encapsulating the nanoscale domains of one liquid within the other. In the synthesis of nanoparticles, this method involves co-precipitation within a reverse micelle, specifically the water-in-oil type. Here, surfactant molecules assemble into a monolayer at the interface, creating a confinement effect that restricts particle nucleation, growth, and agglomeration. The process of preparing IONPs in a microemulsion involves combining a microemulsion containing iron salts with another emulsion comprising precipitating agents. Reactant exchange occurs via diffusion when microdroplets collide. The microemulsion technique boasts various advantages, including its thermodynamic stability and straightforward preparation process [134,148,149,150].

Another possible pathway for the preparation of MNPs is the controlled thermal decomposition of suitable organometallic precursors. In this approach, organometallic compounds such as M(acac) (where acac stands for acetylacetonate), M(oleate), M(cup) (where cup stands for N-nitrosophenylhydroxylamine), or M(carbonyl), which contain metal elements (often transition metals), are thermally decomposed at elevated temperatures. This occurs in the presence of surfactants and organic solvents such as fatty acids, oleic acid (OA), oleylamine (OAm), and hexadecylamine (HDA). In contrast to co-precipitation, thermal decomposition allows for the precise control of particle size, distribution, and shape. However, the use of toxic and expensive precursors and organic solvents at high temperatures, which require a higher energy input, makes this process less environmentally friendly [151]. A key aspect of this method is to achieve a greater separation between nucleation and growth, which is in line with the LaMer theory of nucleation growth. In addition, there are two different methods for producing monodisperse nanoparticles: ‘heating’ and ‘hot injection’. In the heating method, a solution containing all reagents is gradually heated to a certain temperature. In the hot injection method, on the other hand, the reagents are introduced into an already hot surfactant solution, which promotes rapid and homogeneous nucleation [152]. While this method necessitates relatively high-temperature conditions, typically completing the reaction in under 2 h, the nanoparticles produced through thermal decomposition exhibit promising potential for creating MNPs. These nanoparticles can be customized to possess specific component sizes and shapes with a narrow size distribution. Moreover, the resulting nanoparticles commonly feature an organic coating, ensuring colloidal stability in various organic solvents and mitigating oxidation and aggregation tendencies [153,154].

The sol–gel method is based on the hydroxylation and condensation of molecular precursors in a solution, resulting in a ‘sol’ with nanoscale particles. Subsequent condensation and inorganic polymerization steps lead to the formation of a three-dimensional metal oxide network known as a wet gel. The reaction usually takes place at room temperature, so additional heat treatments are required to achieve the final crystalline state. The process involves several successive steps. First, a stable solution of the precursors (the so-called ‘sol’) is formed, followed by gelation triggered by polycondensation or polyesterification reactions that form an oxide- or alcohol-bridged network (the gel). The gel is then aged by further polycondensation reactions, which transform the gel into a solid mass. In this phase, the gel network contracts and displaces the solvent from its pores until the gel is dried, which can lead to the formation of either an aerogel (by supercritical drying) or a xerogel (by thermal evaporation of the solvent). The dehydration of the methanol (MeOH) groups on the surface is usually carried out by calcination at temperatures of up to 800 °C. This final annealing step leads to the aggregation of the nanoparticles [134,151,155].

The solvothermal method, also known as the hydrothermal method when water is used as a solvent, is one of the oldest techniques in ‘green chemistry’ for the effective growth of crystals from various materials. The synthesis process takes place at relatively low temperatures (130–250 °C) so that no additional annealing steps are required. Operation in a closed system prevents the emission of toxic gasses, while the recyclability of unused components and the use of water as a solvent increase the efficiency and environmental friendliness of the process [134,151,156,157].

With this method, large quantities of highly crystalline nanocrystals with well-controlled dimensions can be produced at a low cost. The introduction of microwaves as a heating system has significantly improved the applicability of this synthesis method. Key benefits include fast and uniform heating rates that reduce the process time from several hours to just 30 min, thus reducing the risk of the contamination of the nanoparticles by heating elements and reactor walls. In addition, the precise control of the reaction time makes the method more feasible for large-scale production. Essentially, the process involves a heterogeneous chemical reaction under supercritical or near supercritical conditions in the presence of a solvent maintained above room temperature and at a high pressure. The reactants and solvents used for hydro/solvothermal synthesis vary, including iron complex precursors in high-boiling-point organic solvents and fatty acids or amines as stabilizing agents. Inorganic salts are generally used in aqueous media, while organometallic compounds are preferred in organic solvents. The effective control of the reaction time, temperature, surfactants, and concentrations of starting reagents and solvents used are crucial parameters to achieve the desired crystal structures and sizes of MNPs. This method is particularly versatile in terms of the size and shape of the nanoparticles and allows for the fine tuning of particle sizes within a wide range. In general, longer reaction times lead to larger nanoparticles [145,158].

The functionalization of MNPs is extremely important, as it plays a crucial role in adapting and improving the properties of these nanoparticles for various applications. This process allows for precise control over their physical, chemical, and biological properties so that the nanoparticles can be customized for specific needs in different fields [134,145,158].

The functionalization of MNPs plays a crucial role in medicine, as well as in industry and research. In medicine, functionalized MNPs can be used for targeted drug delivery [159,160], magnetic resonance theranostics [140,149], imaging [161,162], and disease detection [143,144]. They are also essential for the bioanalysis, isolation, and manipulation of biomolecules.

There are two general methods for the surface functionalization of MNPs [163]. The first is in situ surface functionalization, which involves one-pot synthesis. In in situ surface functionalization, functional groups or molecules are added during the synthesis process in which the MNPs are formed. This method allows for control over the properties of the nanoparticles during their formation, which can lead to a more homogeneous and uniform distribution of functional groups on the surface of the nanoparticles. In situ functionalization also provides a better binding of the functional groups to the surface of the nanoparticles, as they are incorporated during synthesis [164,165]. The second method is post-synthetic surface functionalization. In post-synthesis surface functionalization, the MNPs are first synthesized without functional groups on their surface. Later, functional groups or molecules are applied to the surfaces of the existing nanoparticles. This approach allows for better control over the specificity of the added functional groups, so that the surface of the nanoparticles can be customized according to the desired application or function. This method is useful when previously synthesized nanoparticles are to be adapted for a specific application or when it is necessary to functionalize nanoparticles with different types of functional groups [164,165,166,167,168]. Both methods have their advantages and can be used depending on the desired properties and applications of MNPs. In situ functionalization allows for a more uniform distribution of functional groups, while surface functionalization after synthesis offers a greater flexibility in the selection of functional groups for a particular application.

Functionalizing MNPs enables diverse nano-bio applications, facilitating the precise targeting, stabilization, and identification of biochemical entities. A range of materials can be employed to meet the necessary standards for MNP-based nano-biological applications, effectively customizing the surfaces of MNPs [143]. Organic compounds serve as valuable means for functionalizing MNPs, whether during their synthesis or post-synthesis. These nanoparticles exhibit an excellent biodegradability and compatibility due to the coated organic compounds, while retaining fundamental magnetic properties. They are extensively applied in electromagnetic shielding, MRI, magnetic recording, and, notably, in the biological realm for targeted drug delivery and magnetic cell separation. Moreover, organic compounds offer reactive functional groups like carboxyl, amine, hydroxyl, and aldehyde groups [169]. These groups can bind to active sites on biological materials such as DNA, antibodies, proteins, enzymes, etc., broadening their spectrum of biological applications. Furthermore, for enhanced stability and the prevention of MNP agglomeration, various organic compounds such as starch, dextran, poly(ethylene glycol) (PEG), polyethylenimine (PEI), and poly (D, l-lactide) (PLA) are utilized, especially for hydrophilic organic substances [164,167,170].

Inorganic compounds exhibit a variety of properties, such as a strong optical absorption (especially in precious metals such as Au and Ag), high electron density, photoluminescence (as in quantum dots such as CdTe or CdSe), magnetic moment, or phosphorescence (in doped oxide materials such as Y_2_O_3_). These coatings play a crucial role in the stabilization of nanoparticles and are widely used to improve the efficiency of semiconductors, optoelectronics, data storage, quantum dots, catalysis, biological labeling, optical bio-imaging, and in various other fields.

Certain inorganic materials, including silica, metal oxides, Au, carbon, and others, are particularly suitable for binding a range of biological ligands to the surface of Fe_3_O_4_ nanoparticles [171,172,173,174]. However, the identification of a universally suitable ligand for the uniform coating or capping of MNPs remains a major challenge. In addition, precise control over the biocompatibility, shape, structure, stability, and magnetic properties of MNPs using organic materials remains problematic, limiting their use in biological applications. Conversely, capping MNPs with inorganic materials can improve their antioxidant properties compared to bare MNPs, further expanding the scope of nano-biological applications.

The methods of functionalization are extremely diverse. For instance, the chemical functionalization of MNPs involves binding organic molecules such as silanes or other ligands to the nanoparticle surface. These ligands can ensure dispersion stability, prevent agglomeration, and enable further particle modifications [163,164,175].

Moreover, polymer coatings are also utilized. This approach allows for control over particle size, dispersion, and stability, potentially leading to an enhanced biocompatibility and functionality of nanoparticles.

Biological functionalization is yet another crucial method that facilitates the binding of biomolecules such as proteins, enzymes, or DNA to the nanoparticle surface. This approach is highly significant for medical applications, enabling targeted drug delivery and diagnostic use, as well as the development of biological sensors and detection systems [135,174,176,177].

The ability to control and adjust the properties of MNPs through functionalization is fundamental for their successful implementation in numerous applications. It enables their customization to meet specific needs in various fields and applications.

## 7. Published Scientific Articles on the Removal of MPs and NPs Using MNPs

MPs and NPs have been extensively identified in aquatic environments and have emerged as contaminants of increasing concern. There is an urgent need to investigate effective methods for removing MPs and NPs from water. In this section, we focus on research from this field over the past 5 years.

Recently, magnetic separation technologies have attracted considerable attention as a highly efficient method for capturing and removing nanoscale MPs and NPs. These technologies utilize the advantageous combination of a significant active surface area and the ability for rapid and straightforward magnetic recovery from water. They are also inexpensive and available in large quantities [178,179].

Previous research has shown that magnetite-studded technologies play a critical role in promoting the formation of large aggregates of MPs. These aggregates can be efficiently captured and removed from water through the use of magnetic filtration systems.

**Figure 10:**
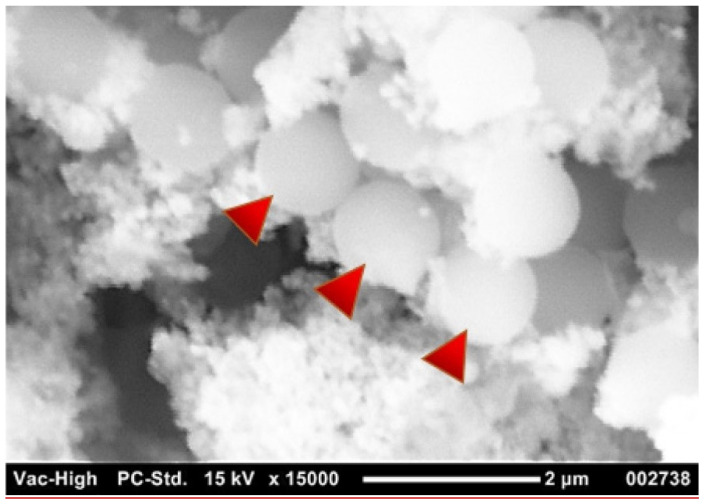
NP particles (red arrows) bound to iron oxide nanoparticles. Taken from [4].

The essential mechanism of interaction between MNP and MPs/NPs seems to be sorption, followed by coagulation and subsequent sedimentation. Detailed studies using iron oxides (magnetite, maghemite, and hematite) have revealed that both chemical bonding and electrostatic interactions between oppositely charged particles play major roles in the bonding, whereby the –OH groups of magnetite seem to enable strong complexation between the MNPs and NPs, while maghemite and hematite attach to NPs only via physisorption [180]. All iron oxides used in the study are capable of removing NPs from aqueous media, with magnetite having the best removal efficiency, presumably due to its highest positive surface charge. The interaction between MNPs and NPs has been studied by Martin et al. [4], pointing out the importance of selecting appropriate hydrophobic coatings in order to achieve the best interaction between MNPs and plastic particles. High–resolution SEM images (Figure 10) included in [4] clearly reveal the presence of multiple NPs particles bound to iron oxide.

When MPs bind to MNPs, either through electrostatic interaction or through specific binding groups, the magnetized MPs can be directly captured under the influence of an external magnetic field [181]. Consequently, magnetic nanotechnologies are considered to be a highly effective approach for the removal of MP contaminants in water.

Table 3 provides a summary of recent research on the removal of MPs and NPs from water samples using MNPs. A multitude of characterization methods have been used during these investigations, including X-ray diffraction (XRD), superconducting quantum interference device (SQUID), attenuated total reflectance Fourier-transformed infrared spectroscopy (ATR-FTIR), X-ray photoelectron spectroscopy (XPS), vibrating sample magnetometer (VSM), ultraviolet–visible spectroscopy (UV-Vis), Brunauer–Emmet–Teller surface area analysis (BET), field emission scanning electron microscopy (FESEM), and energy dispersive X-ray analysis (EDX). The meanings of further abbreviations used in Table 3 have already been explained in the previous chapters.

For example, Yan et al. [182] investigated the influence of particle aggregation behavior in the nanoscale removal of MPs by Fe_3_O_4_ nanoparticles by monitoring the DLS parameters and analyzing the microstructures of particle aggregates. The Fe_3_O_4_ nanoparticles were synthesized by coprecipitation and their performance in the removal of MPs with particle sizes ranging from 100 to 1000 nm was investigated. The aggregation behavior of the particles was determined under different pH and salinity conditions to determine the subsequent effects on the MP removal efficiency. The results showed that from 83.1% to 92.9% of the MPs could be removed by Fe_3_O_4_ nanoparticles within one hour. The results of this study suggest that nanomagnetic separation technologies have a good potential for the efficient removal of MPs from water, which is a critical global problem that threatens both human and ecosystem health.

Shi et al. [20] developed an alternative method for the removal of MPs by magnetic Fe_3_O_4_ nanoparticles. They used Fe_3_O_4_ nanoparticles to magnetize four types of common MP, including PE, PP, PS, and PET with a size of about 200–900 μm, and achieved more than 80%. The removal rate varied depending on the polymer and size of the MP and was positively related to the density of Fe_3_O_4_ NP absorbed on the MP surfaces. In addition, the removal rate of MPs in artificial seawater was relatively high compared to in pure water.

Heo et al. [11] investigated the ability to use magnetic IONPs (Fe_3_O_4_) for the adsorptive removal of micron-sized PS particles. They mixed an aqueous sample of PS particles and IONPs and 1 min was sufficient to effectively remove the PS particles from the water using a magnet. They found that the PS particles were adsorbed on the IONPs and Fe_3_O_4_-PS complexes were formed. It was concluded that the aggregation of iron oxide with PS particles was mainly due to hydrophobic interactions between them. In a real water sample from a river, the adsorption of PS particles on iron oxide particles was inhibited by coexisting ions and suspended solids, but the adsorption efficiency could be improved by higher concentrations of the adsorbent IONPs. The IONPs were released from the Fe_3_O_4_-PS complexes by subjecting the complexes to a simple ultrasonic treatment. They found that the IONPs exhibited good adsorption properties and were effective in removing MPs from the environment.

Martin et al. [4] investigated IONPs with hydrophobic coatings to magnetize plastic particles for removal. They produced and tested IONPs synthesized under airless conditions and in atmospheric air with different hydrophobic coatings based on polydimethylsiloxane (PDMS). They tested the binding and recovery of NP and MP particles from saltwater and freshwater samples. They removed 100% of the particles in a size range of 2–5 mm and almost 90% of the NP particles in a size range from 100 to 1000 nm using a simple permanent magnet (NdFeB). They concluded that IONPs are ideal candidates for water remediation and the removal of a range of compounds of interest, including NPs, via adsorption. IONPs are an environmentally friendly, cost-effective option. They concluded that IONPs are ideal candidates for water remediation and the removal of a number of compounds of interest, including NP, by adsorption.

Gaß et al. [183] investigated the size-dependent water treatment of MPs and NPs with modified SPIONs. They systematically investigated the sizes of MPs and NPs along three orders of magnitude for three different polymers (PS, PMMA, and melamine resin (MR)) and three differently functionalized SPIONs. The SPIONs were functionalized with either n-octadecylphosphonic acid (PAC_18_), (12-dodecylphosphonic acid)-N,N-dimethyl-N-octadecyl ammonium chloride (PAC_12_NC_18_), or 1-methyl-3-(dodecylphosphonic acid) imidazolium bromide (PAC_12_Imida). The remediation efficiency for different sizes (100 nm to 100 μm) of PS, PMMA, and MR was investigated. They found that NPs were collected most efficiently in terms of the number of MPs and NPs collected due to their large specific surface area. In terms of the mass of MPs and NPs removed, MPs between 1 and 5 μm showed the best efficiency in SPION remediation. They also developed a semi-empirical goodness-of-fit model to represent the general trend that emerged for all polymers tested.

Li et al. [184] reported the design and demonstration of a self-driven magnetorobot (SMR) capable of removing and separating MPs/NPs from non-marine waters in a recyclable and scalable manner. SMR consists of an ion-exchange resin microsphere functionalized with SPIONs. They demonstrated the usefulness of SMRs for efficient MP/NP removal, magnetic separation, and the controlled release of MPs/NPs. The broad adaptability of these SMRs was confirmed for plastics with different compositions (PMMA, PA, PS, PVC, and poly(1,1-difluoroethylene) (PVDF)), sizes (200 nm to 40 μm), and surface charge states (positive or negative zeta potential). After ion exchange, the SMRs showed a removal efficiency of over 90% for MPs/NPs in lake water, river, and wastewater and could maintain their performance over 100 treatment cycles.

Li et al. [185] presented a novel approach using magnetic Janus microparticles (MJMs) synthesized via a modified Pickering emulsion method with aminated Fe_3_O_4_@SiO_2_ as the raw material. They investigated the effectiveness of the MJMs in removing PS and PE MPs from water. They used paraffin as a masking agent and PAC_18_ as a grafting material for MJM preparation. The resulting particles exhibited a characteristic asymmetric flower-shaped structure on the surface. The MJMs showed an exceptional efficiency in the adsorption of MPs. At an MP suspension concentration of 2 mg/mL and an adsorbent dose of 1 mg/mL, the MJMs achieved a removal efficiency of 92.08% for PS and 60.67% for PE in just 20 min of contact time. They found that the effectiveness of the adsorption process was due to several factors, including hydrophobic interactions, cation-π interactions, electrostatic attraction, and the efficient dispersion of the particles in water, as shown by analyzing the size distribution and zeta potential. Kinetic and isothermal modeling underpinned the excellent adsorption rate and capacity of the MJMs for MPs.

Wang et al. [186] prepared Fe_3_O_4_ superhydrophobic magnetic adsorbents (Fe_3_O_4_@C_n_, n = 12, 14, 16, 18) modified with various saturated fatty acids (C_12_, C_14_, C_16_, and C_18_) by liquid phase deposition. It was shown that Fe_3_O_4_@C_n_ had the properties of magnetic materials with superhydrophobic properties and, thus, had the ability to separate oil and water. In the removal of MPs in five liquid food systems, Fe_3_O_4_@C_12_ exhibited an adsorption efficiency of 92.89%, which was attributed to the electrostatic and chemical bonding interactions between the MPs and Fe_3_O_4_@C_n_, as suggested by density functional theory (DFT) calculations. Analysis of the Langmuir model with monolayer adsorption revealed that PS had a maximum adsorption capacity of up to 809.29 mg/g and the PS removal process involved both exothermic reactions and chemisorption.

Zhang et al. [187] prepared three surface-modified nano-iron oxide materials (PEI/Fe_3_O_4_, citric acid- CA/Fe_3_O_4_, and polyethylene glycol- PEG/Fe_3_O_4_) and attempted to determine and compare their adsorption efficiencies on PE with different sizes. The results showed that all materials had a good adsorption efficiency on PE, ranging from 68.67% to 96.67%. Nano-sized PEG-modified Fe_3_O_4_ showed a better PE removal capacity for small-sized MPs. This could be related to the amphipathic properties of high-molecular-weight PEG. PEG is also rich in hydroxyl groups and easily forms hydrogen bonds with PE. PEG/Fe_3_O_4_ retained a high adsorption capacity even at low temperatures, while a neutral pH was favorable for the adsorption of MPs. The presence of anions (Cl^−^, SO_4_^2−^, HCO_3_^−^, and NO_3_^−^) and humic acids inhibited the adsorption of MPs. They concluded that the adsorption process was mainly driven by intermolecular hydrogen bonds and that PEG/Fe_3_O_4_ is an effective means of combating MPs.

Babalar et al. [188] synthesized a magnetically activated biochar–zeolite composite coated with PEG and PEI and improved its electrostatic properties for the adsorption of 2 μm and 15 μm MPs. They used various characterization techniques to verify that the materials had been successfully synthesized. Applying Fe_3_O_4_ and zeolite to the biochar surface increased the equilibrium adsorption of MPs from 72.9 and 84.3 mg/g in activated biochar to 96 and 100 mg/g in magnetically activated biochar zeolite. To evaluate the effects of pH and temperature on adsorption, response surface analysis (RSM) was performed using a centralized composite design. The optimization results showed a pH of 4 and temperatures of 28 and 24 for the maximum adsorption of 2 μm and 15 μm MPs, respectively. They found that the produced material could be regenerated for at least four cycles.

Tang et al. [189] were the first to investigate the removal of MPs using magnetic carbon nanotubes (M-CNTs) as adsorbents. Their study aimed to synthesize efficient and recyclable M-CNTs for MP removal, optimize the operating conditions for the recycling and reuse of M-CNTs, and investigate the mechanism of the M-CNTs-based MP removal process. Absorption with M-CNTs was effective for PE, PET, and PA, and all MP/M-CNTs composites were separated from aqueous solutions by magnetic force within 300 min. The mechanism analysis clearly indicated that the adsorption of M-CNTs by PE was caused by the strong hydrophobicity of MP, the adsorption of M-CNTs by PET was caused by hydrophobic interaction and π-π-electron conjugation, and the π-π-electron interaction, complexation, electrostatic interaction, and hydrogen bonding interaction on the PA surface contributed to the adsorption of M-CNTs. They showed that the used M-CNTs could be recycled by thermal treatment at 600 °C and the recycled ones (up to four times) could still be used to remove <80% of the MP.

Zhou et al. [130] prepared a CuNi carbon material (CuNi@C) by a hydrothermal method to remove PS NPs from water. They investigated the adsorption performance, kinetics, isotherms, thermodynamics, and electrostatic attraction during the adsorption process. The main objectives were to synthesize efficient and reusable CuNi@C for the removal of PS NPs, explore the adsorption performance of CuNi@C and the mechanism of PS NPs removal, and study the effects of ions on the removal efficiency. The results showed that when the CuNi@C dosage was increased from 0.1 to 0.3 g/L, the removal efficiency of PS NPs (10 mg/L) increased from 32.72% to 99.18%. The thermodynamic analysis showed that the adsorption of PS NPs on CuNi@C was a spontaneous and endothermic process. Electrostatic attraction occurred during adsorption, and the removal efficiency of PS NPs in the acidic system was generally higher than that in the alkaline system. CuNi@C could be recycled by washing and drying, and after four cycles, CuNi@C could still remove about 75% of the total PS NPs from the water.

Li et al. [190] investigated, for the first time, the interactions between Ag nanoparticles (AgNPs) and PE, PP, and PS MPs in aquatic environments. Their main aim was to investigate the interactions between AgNPs and PE, PP, and PS MPs to evaluate the colloidal stability of AgNPs when encountering MPs and explain the interaction mechanisms between the pollutants. Their results showed no significant interactions between the AgNPs and PE or PP MPs, but the AgNPs were efficiently removed by PS MPs. They attributed these differences to the presence of π-π interactions. AgNPs were significantly trapped on the surface of the PS MPs in the form of Ag^0^ rather than Ag^+^. They found that the trapping process was a monolayer adsorption and was strongly influenced by the mass ratio of AgNPs and PS MPs. These results demonstrate the complexity of the adsorption of AgNPs on MPs and improve the current understanding of the interactions between nanoparticles and MPs in the aquatic environment.

Zhang et al. [191] prepared a magnetic magnesium hydroxide coagulant (MMHC) by adding magnetic Fe_3_O_4_ particles during the formation of Mg(OH)_2_ to remove PE from wastewater, which floats easily on the water surface and is a major component of MPs. They compared the efficiency of MP removal after coagulation with the conventional magnesium hydroxide coagulant (MHC). They prepared three MMHCs by changing the ratio of Mg^2+^ to OH^−^ in the formation of magnesium hydroxide. The results showed that, among the three types of MMHC, when the ratio of Mg^2+^:OH^−^ was 1:1, the highest MP removal efficiency of 87.1% was achieved, which was 14.7% higher than that of MHC alone. They found that the removal efficiency of MPs did not change significantly in the pH 5–9 range and charge neutralization occurred during the coagulation process. The particle size of the two types of coagulants shifted from large to small with aging, while the flocs produced by the two coagulants showed an opposite trend.

Zhang et al. [192] synthesized a magnetic material CuFe_2_O_4_ for the removal of MPs with different degrees of photoaging. CuFe_2_O_4_ had an excellent property in removing PS MPs and its superparamagnetic nature ensured its magnetic recovery. They aimed to find an efficient and rapid method to quantify PS microspheres (PSMPs), investigated the removal performance of PSMPs with different photoaging degrees on CuFe_2_O_4_, discussed the effect of environmental factors on PSMP removal, and clarified the mechanism of FeCu_2_O_4_ in the removal of PSMP. At a CuFe_2_O_4_ dosage of 0.2 g/L, removal efficiencies of 98.02, 94.07, and 96.93% were achieved for the original, 36 h-, and 84 h-aged PSMPs with an initial concentration of 0.04 g/L, respectively. In addition, a high removal efficiency was also achieved in current water. The effects of different environmental factors on the removal efficiency were in order of dissolved organic pollutants > pH > salt ion concentration. Hydrogen bonding played a key role in the removal of pristine PSMPs, and the destruction of C=O by Fe-OOH also played an important role in the removal of aged PSMPs. Pyrolysis at 500 °C for 4 h could generate it, and the removal efficiency for PSMP could still reach 83.88% after four times of reuse.

Zhao et al. [193] used PSU (FA) as a substrate to synthesize a new magnetized material (NMA) by the one-step coprecipitation method, which is a simple preparation method, and the synthesized material had a high adsorption capacity. They aimed to explore the removal capacity of Fe-modified FA on PS NPs (PSNPs), investigate the interfering effect of different environmental factors on the removal of PSNPs in aquatic environments, and study the possible interaction mechanisms between Fe-modified FA and PSNPs through different characterization methods. The different characterization analyses revealed the strong interaction between NMA adsorbents and PSNPs and showed that the PSNPs were successfully bound to the surface and pores of the material. The pH range between weak acidity and neutrality was favorable for the adsorption of PSNPs, and the adsorption amounts of PSNPs were 82.8–89.9 mg/g at pH 5–7, confirming that electrostatic attraction, complexation, and π-π interactions were involved in the adsorption process. The adsorption/desorption experiment showed that the NMA adsorbents had an excellent reusability for PSNPs; they could be used four times.

Pasanen et al. [194] synthesized a zeolitic imidazolate framework (ZIF-8), a magnetic porous nanocomposite modulated with n-butylamine (nano-Fe@ZIF-8) in water at room temperature. Their aim was to develop the first magnetic porous nanomaterial based on a previously unknown combination of an organic (n-butylamine) and an inorganic modulator (Fe^2+^) through the aqueous synthesis of ZIF-8 (nano-Fe@ZIF-8) at room temperature. The performance of the resulting magnetic porous nanocomposite was evaluated for the removal of MPs and endocrine-disrupting phenols from aqueous samples. The prepared nano-Fe@ZIF-8 enabled the rapid and simultaneous removal of both PS microspheres (1.1 μm diameter) and endocrine-disrupting phenols (bisphenol A and 4-tert-butylphenol). Nano-Fe@ZIF-8 showed a higher removal efficiency compared to unmodulated Fe@ZIF-8. Under optimal conditions, nano-Fe@ZIF-8 (20 mg) could remove ≥98% of the PS microspheres at a high concentration (25 mg/L) within 5 min, and ≥94% of bisphenol A (1 mg/L) and 4-tert-butylphenol (1 mg/L) within the same time frame. Nano-Fe@ZIF-8 showed a comparable PS microsphere removal efficiency and greatly improved extraction performance for two selected endocrine-disrupting phenols compared to Fe_3_O_4_ MNPs functionalized with OA and azelaic acid. They concluded that the results showed that the synthesis was simple and environmentally friendly and that they synthesized a high-performance material for the rapid removal of soluble organic pollutants and microparticulated organic pollutants.

Surette et al. [195] utilized NP particles consisting of a polyacrylonitrile (PAN) core with a trace metal label (palladium [Pd]) and a PS shell (PAN-Pd@PS NPs). Hydrophobically functionalized MNPs (HDTMS-FeNPs) were used as part of a method for separating and concentrating NPs from environmentally relevant matrices PAN-Pd@NPs to enable the low-level detection and validation of the separation technique. PAN-Pd@NPs were recovered from ultrapure water, synthetic freshwater with a model isolate of natural organic matter, and from synthetic seawater, with recovery rates for PAN-Pd@NPs of 84.9%, 78.9%, and 56.1%, respectively. In the initial tests of the method, they found that the addition of NaCl in ultrapure water, synthetic freshwater, and synthetic freshwater with a natural organic matter (NOM) model was required to cause the aggregation and attachment of the particles. They concluded that MNPs in combination with a flow-through system are a promising technique for the extraction of NPs from aqueous suspensions with different compositions.

Oliva et al. [196] synthesized magnetic bismuth ferrite (BiFO) microparticles for the removal of PS NP/MPs from drinking water. BiFO consisted of porous agglomerates with a size of 5–11 μm, while the PS NP/MPs had sizes in the range of 70–11,000 nm. The PS NP/MPs were dispersed in water, then BiFO microparticles were added to the contaminated water, and later, the mixture of BiFO and PS particles was irradiated with near infrared (NIR) light (980 nm). Subsequently, the BiFO particles covered with PS NP/MPs were separated from the water using a neodymium magnet. After applying this last procedure, the PS NP/MPs were removed from the drinking water at pH = 7 with an efficiency of 100%. When no NIR light was used, the removal efficiency dropped to 4.7%. It was found that the BiFO microparticles with NIR light had two main advantages: it melted the PS NP/MPs on the BiFO surfaces and it induced the formation of oxidizing species, which, in turn, strongly degraded the by-products formed in the water, so that a very high value for a total organic carbon (TOC) removal of 95.5% was achieved. The PS NP/MPs were removed by electrostatic attraction, which contributed to the removal of PS from the drinking water. They concluded that the results showed, for the first, time that porous BiFO microparticles can be used for the magnetic removal of PS from water, and that the technique demonstrated here could also be applied to the removal of other plastic contaminants.

Bakhteeva et al. [197] prepared composite magnetic Fe-C-NH_2_ MNPs with a core–shell structure by the gas condensation method, followed by a reaction with the reaction with the diazonium salt. The as-prepared MNPs were used for the removal of PE and PET MPs from model aqueous suspensions by magnetic sedimentation of the formed heteroaggregates in a magnetic field produced by permanent magnets. Considerably different results were obtained for both types of MPs: while the magnetic sedimentation efficiency for PET was close to 100% after 15 min of sedimentation, it was only 88% for PE at the same reaction conditions. In order to increase the efficiency of PE removal, longer sedimentation times or an increase in the concentration of MNPs had to be used. The difference could be at least partly attributed to the higher hydrophilicity of PET NPs, allowing for a more active attachment of MNPs when compared to PE.

Peng and coworkers [198] used the naturally occurring algae *C. vulgaris* to prepare low-cost biohybrid microrobots decorated with Fe_3_O_4_ NPs on the surface. The surface decoration allowed for precise actuation and manipulation of the microrobots, instead of the uncontrollable Brownian motion exhibited by bare algae cells. The active motion of the as-prepared microrobots allowed for a considerable efficiency increase (up to 92%) in the removal of amino-modified polystyrene MPs and NPs from model suspension, when compared to static conditions (41%). The microrobots also showed considerable promise in removing MPs and NPs when tested in real aquatic environments.

Although the subject highlighted in our review paper is very popular and has been under intense investigation in recent years, many challenges remain which need to be resolved before the removal of MPs and NPs using magnetic nanoparticles can become viable and suitable for usage in large-scale processes. Nanomaterials based on iron oxides, being environmentally friendly and affordable, seem to be ideal candidates for water remediations. However, much additional work will be needed to optimize the MNP size, preparation methods, and to develop the most appropriate coatings in order to achieve the optimal removal efficiency. Further research will also be required to develop suitable methods for the large-scale magnetic separation of plastics/MNP agglomerates from water, the recycling of MNPs, and finally, to resolve the question of dealing with MPs and NPs collected from aqueous systems. In addition to the purely scientific point of view, a careful cost analysis of different remediation approaches will be crucial before practical implementation of the method.

## 8. Conclusions

The widespread use of plastic in today’s world, covering all aspects of our lives—from packaging our food to constructing our vehicles—is causing significant environmental and health consequences. The widespread presence of MPs and NPs in the air, soil and—arguably most pressing—in aquatic environments like rivers, oceans, and marine sediments has become the subject of intense investigations during the last decades. At the beginning of the review, we summarized the sources of plastics in the environment and discussed various methods for the detection of MPs and NPs. Later on, we provided an account of properties of MPs and NPs in marine environments and their impacts on the environment and human health. A major part of the review is oriented towards a thorough overview of techniques, which are either already used or under intense investigation for removing MPs and NPs from water. Many traditional methods for drinking water treatment achieve satisfactory results for the removal of MPs, while finding suitable methods for the efficient removal of NPs remains a challenge.

Iron-based magnetic nanoparticles (MNPs), especially magnetite, maghemite, and ferrites, can provide an ideal platform for the effective removal of MPs and NPs from water due to their biocompatibility, low cost, and sustainability [199]. We provide a thorough summary of the different methods used for MNPs synthesis, each of them offering its own advantages and disadvantages. The most widely used among them seem to be co-precipitation, microemulsion synthesis, the thermal decomposition of organometallic precursors, sol–gel method, and solvothermal/hydrothermal preparation. Since the functionalization of as-prepared MNPs plays a crucial role in ensuring stability and preventing oxidation and aggregation, we presented both major surface functionalization methods (in-situ and post-synthetic approach) and summarized the materials employed for functionalization. A summary of recent papers, reporting the removal of MPs and NPs using MNPs, is provided in the final part of the review, summarizing their removal efficiency and pointing out the main findings. The review should inspire researchers in further studies, leading to more efficient and sustainable water treatment systems and enabling the upgrading of existing wastewater treatment plants, with the ultimate goal of responsible plastics use while minimizing their effect on the environment [200,201].

## Figures and Tables

**Table 1:** Certain characteristics of some MPs in real samples.

Place	MPs Type	MPs Size	Profusion
The Netherlands (tap and surface water)	PS, PE	50, 100, 200, 500, 1000 nm (PS), 90–106 µm	260 mg/L
Denmark (fish sample)	PS, PE	100 nm (PS), 200–9900 nm (PE)	1.3 mg/g fish
USA (seawater)	PP, PE, PS, PA	≤5 mm	0.025 g/mL
South Korea (seawater and beach)	Expanded polystyrene (EPS)	≤1 mm	0–0.3 items/L (seawater), 631 items/L (beach)
China (surface sediment)	Rayon, PE, PP, PA, PET, PS, PMMA, PU	34.97–4983.73 μm	499.76 items/kg
Italy (shallow waters)	PE, PP, PS	≤1 mm	672–2175 items/kg
China (sediment)	HDPE, PET, PE, PS	≤5 mm	5.1–87.1 items/g sediment
China (sewage)	PET, PS, PP	681.46 ± 528.73 μm	0.59–12 items/L
China (freshwater bodies)	PES, rayon, PP, PA, nylon	20 to 5000 μm	0.9–2.4 items/L
Australia (shrimp)	PS, rayon	0.190–4.214 mm	0.40 ± 0.27 items/L
China (fishes)	PE, PP, PES	20–500 μm	0.3–5.3 items per fish
Germany (bottled water)	PET, PP	1–500 μm	0–253 items/L
Mexico (milk)	PES, polysulfone (PSU)	≤5 mm	3–11 items/L

**Table 2:** Comparison of some mentioned methods with their parameters, efficiency, and limitations (summarized by [3]).

Method	The Size of Removed NP [nm]	Removal Efficiency [%]	Limits
Filtration	217–333	32–92	Not suitable for larger particles, as they may remain in the fraction.
Ultrafiltration	≤150	74	Particles can evade treatment; the process can be time-consuming.
Flocculation	217	77	More studies are needed to determine the optimal parameters.
Centrifugation	206	98	Time-consuming process.
Photocatalytic degradation	≤100	17.1	The phototransformation of NPs can vary and the photo-reactive activity in water can be high.
Membrane bioreactor filtration	≥2	99	Proper hydraulic retention time.

**Table 3:** A summary of sampling, processing, characterization methods, removal efficiency, and adsorption capacity of MPs and NPs using MNPs.

Sample Types	MNPs	Characterization	Method	Particles Size of MPs, NPs	Removal Efficiency/Adsorption Capacity	Main Findings	Ref.
Solutions of MP (PS)	Fe_3_O_4_	XRD, FTIR, DLS, SEM, TEM	Magnetic separation	100, 500, 1000 nm	83.1–92.9% in a 1 h period	Electropositive Fe_3_O_4_ MNPs and electronegative MPs efficient removal of MP.	[182]
Solutions of MPs (PE, PP, PS, PET) in pure, artificial, and environmental water samples	Magnetic nano-Fe_3_O_4_	FTIR, SEM	Surface absorption, magnetic separation	200–900 μm	62.83–86.87% in a max. 240 min	Properties of MPs such as crystallinity, hydrophobicity, and density influence the removal efficiency.	[20]
microPS particles from the water	Magnetic iron oxide (Fe_3_O_4_) nanoparticles	TEM, FTIR	Adsorption process, desorption process	0.08, 0.43, 0.7 and 1 μm	42.0–93.7% depending on the concentration, total surface area and number of PS particles	Hydrophobic interactions are the main interactions involved in the aggregation of Fe_3_O_4_ with PS particles.	[11]
Salt and fresh water samples	Iron oxide nanoparticles with several polydimethylsiloxane hydrophobic coatings	SEM, TEM, SQUID, DLS, XRD, zeta potential	Absorption process	2–5 mm; 100–1000 nm	90.0–100%	Removed 100% of from 2–5 mm, and nearly 80% of NP particles from 100 nm to 1000 nm.	[4]
Solution of PS, PMMA, ME	Modified superparamagnetic γ-Fe_2_O_3_, 9.6 nm	ATR-FTIR, TGA, DLS; SEM,	Magnetic removal	100 nm–100 μm	Polymer types of 2.5–5 μm for the maximum removal yield in terms of removed MPs and NPs mass (up to 5.38 g/g SPION); MPs and NPs of 100 nm–1 μm in terms of highest numbers (up to 10 trillion MP and NP fragments per gram SPIONs)	If the size of the MPs is further increased, number as well as mass related efficiency is reduced as the specific surface area decreases rapidly.	[183]
Nonmarine waters in a recyclable and scalable way	SMR consists of an ion-exchange resin microsphere functionalized by superparamagnetic Fe_3_O_4_ nanoparticles	SEM, EDX, magnetic measurements, DLS, a confocal microscope	Dynamic adsorption process; magnetic removal	0.2–40 μm	>90% over 100 treatment cycles	The magnetorobot shows sustainable removal efficiency of >90% over 100 treatment cycles.	[184]
Solutions of MPs (PS, PE)	Magnetic Janus microparticles (MJMs) synthesized via a modified Pickering emulsion method with aminated Fe_3_O_4_@SiO_2_ as the raw material	FTIR, TGA, SEM, contact angle analysis	Adsorption process	10 μm	92.08% for PS and 60.67% for PE in just 20 min	Kinetic and thermodynamic studies confirmed the remarkable rate and capacity of the MJMs.	[185]
MPs in five liquid food systems	Fe_3_O_4_@C_n_ (n = 12, 14, 16, 18), modified by different saturated fatty acids (C_12_, C_14_, C_16_, C_18_)	TEM, AFM, FTIR, XRD, XPS, VSM, BET, TGA, contact angle measurements	Nitrogen adsorption measurements	100 nm	Fe_3_O_4_@C_12_ exhibited 92.89% adsorption efficiency	Fe_3_O_4_@C_12_ showed the desired adsorption efficiency.	[186]
PE MPs in water	Fe_3_O_4_; PEG/Fe_3_O_4_; PEI/Fe_3_O_4_; CA/Fe_3_O_4_	FTIR, BET, zeta potential analysis, XRD	Magnetism adsorption	13–149 μm	2202.55 mg/g	The PEG/Fe_3_O_4_ exhibited a high magnetic capture efficiency of PE MPs in water.	[187]
PS MPs	magnetic activated biochar-zeolite composite (MACZ) coated with PEG and PEI (PMACZ)	SEM, EDX, BET, XRD, TGA, VSM	Adsorption	2 and 15 μm	736 mg/g and 769 mg/g for PMACZ on 2 μm and 15 μm MP	The efficiency and high cycling capacity of these adsorbents.	[188]
PE, PET, PA	Magnetic carbon nanotubes (M-CNTs)	UV-Vis, VSM, XRD, SEM, FTIR, XPS, zeta potential, TGA	Magnetic force	48 μm	100%	The adsorption of M-CNTs by PE -strong hydrophobicity of MPs, the adsorption of M-CNTs by PET–hydrophobic interaction and π-π electron conjugation, and π-π electron interaction.	[189]
PS NPs in water	CuNi carbon material (CuNi@C)	SEM, FTIR, XPS, XRD, BET	Adsorption process	100 nm	99.18%	After 4 cycles, CuNi@C can still remove ~75% of total PS NPs from water.	[130]
PE, PP, and PS in aquatic environments	Ag nanoparticles	UV-Vis, DLS, TEM, XRD, SEM, EDX	Adsorption process	0.2–0.25 mm	94.52%	Ag nanoparticles could be captured on the surface of PS MPs but coexisted with PE and PP MPs in water solutions.	[190]
PE in wastewater	magnetic magnesium hydroxide coagulant (MMHC) through the combination of Mg(OH)_2_ and Fe_3_O_4_	SEM, FTIR, XRD, zeta potential	Adsorption process	≤270 μm	73.4–92.6%	Removal is the highest when the ratio of Mg^2+^ to OH^−^ reaches 1:1.	[191]
PS MPs in water	CuFe_2_O_4_	XRD, VSM, BET, FTIR, SEM, EDX, XPS,	Remove MPs with different photoaging degrees	0.96–1.59 μm	98.02%	Hydrogen bonding played a key role in the removal of pristine PS MPs and the destruction of C=O by Fe-OOH.	[192]
PS NPs in aqueous solutions	Fly ash modified with Fe ions	UV-Vis, FTIR, SEM-EDX, XRD, XPS, VSM	Adsorption process		94.1%	Fly ash modified with Fe ions adsorbents has excellent reusability for PS NPs.	[193]
PS MPs	A zeolitic imidazolate framework (ZIF-8) magnetic porous nanocomposite modulated with n-butylamine (nano-Fe@ZIF-8)	SEM, XRD, FESEM, BET, nitrogen adsorption-desorption measurements	Magnetic removal	1.1 μm	≥98%	The results illustrate the synthesis of a simple, environmentally friendly and high performing material for the fast removal of both soluble organic pollutants and microparticulated organic pollutants.	[194]
Metal-doped PS NPs in ultrapure water, synthetic freshwater, synthetic freshwater with a model natural organic matter isolate and synthetic marine water	Hydrophobically functionalized magnetic nanoparticles	EDX, DLS, SEM, XRD,	Magnetic separation flow cell	229 nm	56.1–84.9%	MNPs in combination with a flow-through system is a promising technique to extract NPs aqueous suspensions with various compositions.	[195]
PS NP/MPs in drinking water	Magnetic bismuth ferrite (BiFeO) microparticles	XRD, zeta potential measurements,		70–11,000 nm	≈95.5% in 90 min	Using photocatalysis + physical-adsorption is a feasible strategy to quickly remove MPs contaminants from the water.	[196]
Polyethylene (PE) and PET MPs from model aqueous suspensions	Composite magnetic Fe-C-NH_2_ MNPs	DLS, SEM, optical microscopy, XRD, UV-Vis	Magnetic sedimentation	5–30 μm	>99%	PE and PET MPs can be effectively separated from water by adding Fe-C-NH_2_ MNPs.	[197]
Amino–modified PS in aqueous suspension	Magnetic algae robots (algae cells with Fe_3_O_4_ bound on its surface)	SEM, EDX, XRD, zeta potential, magnetic measurements, fluorescence intensity measurements.	Removal under rotating magnetic field	50 nm and 1.5 μm	70–92%	Magnetic field driven algae-mased microrobots can be used for effective capture and removal of micro/nanoplastics from the aquatic environment.	[198]
